# Combination of Cold Helium Plasma with Fluoride Varnish to Improve Enamel Surface Protection

**DOI:** 10.3390/ma18194466

**Published:** 2025-09-25

**Authors:** Sara Fathollah, Hossein Abbasi, Mohammad Sadegh Ahmad Akhoundi

**Affiliations:** 1 Nalecz Institute of Biocybernetics and Biomedical Engineering, Polish Academy of Sciences, Trojdena 4, 02-109 Warsaw, Poland; sfathollah@ibib.waw.pl; 2 Department of Energy Engineering and Physics, Amirkabir University of Technology, Tehran 1591634311, Iran; 3 Department of Orthodontics, School of Dentistry, Tehran University of Medical Sciences, Tehran 1439955991, Iran; dr.s.akhoundi@gmail.com; 4 Dental Research Center, Dentistry Research Institute, Tehran University of Medical Sciences, Tehran 1417614411, Iran

**Keywords:** cold atmospheric plasma, bovine enamel, fluoride varnish, microhardness, SEM/EDX

## Abstract

This study aimed to determine the optimal application sequence of cold atmospheric helium plasma (CAP) with fluoride varnish to enhance enamel protection and fluoride uptake. A total of 91 bovine incisor teeth were randomly assigned into seven groups (*n* = 13 each): negative control (C, no treatment), comparative controls [helium gas (He, gas only)], helium plasma (P, plasma only)], positive control [fluoride varnish (V)], and three experimental groups: plasma followed by varnish (PV), varnish followed by plasma (VP), and plasma before and after varnish (PVP). Specimens were analyzed using scanning electron microscopy (SEM) and energy-dispersive X-ray spectroscopy (EDX), and microhardness testing at 0, 24, and 48 h post-treatment. SEM revealed that helium plasma treatment enhanced the even dispersion of fluoride and reduced imperfections on the enamel surface. EDX analysis indicated significant alterations in the elemental composition, particularly with respect to the amount of fluoride (F) and the calcium-to-phosphorus (Ca/P) ratios. In the PVP group (CAP before and after varnish), the fluoride atomic percentage increased notably from 1.21% (varnish group) to 7.31% at 48h. Concurrently, the Ca/P ratio increased from 1.95 to 2.39 corresponding with a statistically significant 24% improvement in enamel hardness (repeated-measures ANOVA with Bonferroni correction, *p* < 0.05). The timing of CAP application critically affects fluoride absorption and enamel hardening. This study clearly demonstrates how sequential CAP treatment maximizes fluoride effectiveness, offering a promising route for non-invasive caries prevention.

## 1. Introduction

Dental caries is a multifactorial disease mediated by dental plaque (biofilm) and strongly influenced by dietary sugars, oral hygiene, host factors, and the oral microbiome [1,2,3]. While acid production by bacterial metabolism plays a central role in enamel and dentin demineralization, additional factors, including dietary acids, poor oral hygiene, and frequent use of bleaching agents, can accelerate disease progression [4,5]. Conversely, natural remineralization allows daily restoration of enamel via mineral absorption, and prioritizing remineralization over demineralization represents the most cost-effective strategy for caries prevention.

The incorporation of fluoride is widely recognized as a crucial intervention in minimizing the incidence and severity of dental caries [6]. Fluoride ions replace hydroxyl groups in hydroxyapatite, forming fluorapatite, enhancing enamel resistance, and reducing plaque carcinogenicity [7,8]. Systemic and topical fluoride administration are possible, with topical application preferred post-eruption to reduce fluorosis risk [9,10,11].

The application of fluoride varnish, with its appropriate concentration of fluoride ions, offers a safe method for preventing tooth decay. Applying a thin layer of fluoride varnish to the tooth surface is the most straightforward, convenient, and effective form of fluoride administration, surpassing other methods such as fluoride gel, toothpaste, and mouthwash [12,13,14]. Fluoride varnish, typically containing 22.6 mg/mL fluoride in an alcohol-resin medium, adheres to enamel, gradually releasing ions over time. Regular application every 3–4 months is effective, though cost and time remain limiting factors [15,16]. Enhancing varnish retention and fluoride absorption without increasing dosage is therefore desirable.

Cold atmospheric pressure plasma (CAP) has recently emerged as a non-invasive method to modify surface properties, including adhesion, via active particles, free radicals, and ions generated through electric discharge [17,18,19]. CAP offers high surface adhesion, tissue safety, cost-effectiveness, and accessibility, and has been applied in dentin bonding, bactericidal treatments, and oral wound healing [20,21,22,23,24,25,26,27]. Studies demonstrate CAP’s capacity to disinfect dental surfaces [28], enhance osteoblastic proliferation [29], and improve material hydrophilicity without altering intrinsic properties [30].

A study by Kim et al. (2018) aimed to improve fluoride absorption using cold plasma. The findings showed that combining CAP helium with fluoride varnish enhanced fluoride levels in tooth enamel and increased resistance to demineralization compared to fluoride treatment alone [31]. Bapat et al. (2022) further showed that ErCr lasers and CAP significantly improved enamel calcium-to-phosphorus ratios across different varnishes, strengthening enamel [32].

Our previous pilot study examined CAP types (helium, argon, and dielectric barrier discharge) for their effects on varnish adhesion, fluoride absorption, and enamel morphology. CAP helium yielded optimal outcomes with minimal side effects [33]. However, despite these advances, previous studies mainly investigated CAP as a binary intervention (applied or not applied) without systematically evaluating the sequence of CAP relative to fluoride varnish application, nor the short-term temporal dynamics of fluoride uptake (0, 24, 48 h). These aspects remain a critical knowledge gap for optimizing clinical protocols. Therefore, the present study specifically investigates not only whether CAP enhances fluoride varnish effectiveness, but also when and in what sequence it should be applied to maximize enamel protection in real clinical settings. Consequently, the specimens were divided into seven groups: negative control (C, no treatment), comparative controls (He-gas, He-plasma), positive control (V, fluoride varnish only), and three experimental combinations (PV, VP, and PVP). Specimens were analyzed by scanning electron microscopy (SEM), energy-dispersive X-ray spectroscopy (EDX), and microhardness tests.

## 2. Materials and Methods

### 2.1. Specimens Preparation

A total of 91 specimens of recently extracted bovine incisor teeth from an abattoir were used. All procedures were approved by the Tehran University of Medical Sciences Research Committee (IR.TUMS.VSR.REC.1397.757). The bovine teeth were collected from the Tehran Slaughter Centre. Each specimen was processed as described below. To preserve moisture and prevent secondary contamination, healthy teeth were isolated and immersed in a 0.1% chloramine solution at 4 degrees Celsius for four days. After the soft tissues surrounding the teeth were meticulously separated, a slurry-water mixture of pumice powder was used to polish the teeth. To ensure the cleanliness, absence of structural defects, and microfractures of all specimens, a 10× stereo microscope (ZSM1001 model, Zist Rahe Danesh Company, Tehran, Iran) was used to examine the outer buccal enamel surfaces of all specimens. As a result, unsuitable specimens were eliminated. The teeth were precisely cut at the cement-enamel junction using a low-speed diamond saw (Elec-Micromotor MARATHON ESCORT II, Saeshin Precision Co., Ltd., Daegu, Republic of Korea). Enamel pieces were embedded in acrylic resin (Cold-Cure Acrylic for Repair, Acropars company, Tehran, Iran), with the buccal surface oriented upwards. A flat enamel surface was prepared by sequential polishing with 600, 1200, and 1800 grade silicon carbide papers, in order to standardize the surface and ensure more consistent and reproducible measurements. An area of 2 × 6 square millimetres was marked with a graphite pencil on the tooth surface. Lastly, all tooth surfaces were meticulously coated with acid-resistant varnish in two stages, except the designated 2 × 6 square millimetre area. Each specimen was stored individually in distilled water in a sealed container, labelled with a randomized code, and then assigned to the respective experimental groups for subsequent treatments and analyses. Between experiments, the specimens were securely stored in a sealed container with distilled water and maintained at room temperature with a humidity level of 95%.

### 2.2. Specimens Grouping

After completing all preparation steps, the 91 specimens were randomly assigned to seven groups (*n* = 13 each), with the sample size determined in consultation with a biostatistics expert. The study investigated the effect of cold atmospheric helium plasma on enamel surface properties in combination with fluoride varnish. The groups were categorized as follows: negative control (C, no treatment), comparative controls [helium gas (He, gas only), Helium plasma (P, plasma only)], positive control [fluoride varnish (V)], and three experimental groups: plasma followed by varnish (PV), varnish followed by plasma (VP), and plasma before and after varnish (PVP) (Table 1). All treatments were performed once on day 0. Subsequent analyses were carried out at three intervals: immediately after treatment (~1 h, designated as 0 h), 24 h, and 48 h. Microhardness and EDX analyses were performed at all three time points, whereas SEM evaluation was conducted only at the 24 h time point.

### 2.3. Topical Fluoride Application

Among the seven groups, three control groups did not receive fluoride varnish: negative control (C, no treatment), comparative control (He, helium gas only), and comparative control (P, helium plasma only). In contrast, the enamel surfaces of the remaining four groups (V, PV, VP, and PVP) were treated with a 5% sodium fluoride varnish (Oratek, San Diego, CA, USA) using micro brushes to an approximate thickness of 1.38 mm. After application, the specimens were exposed to dry air for two minutes in accordance with the manufacturer’s instructions. The purpose of this was to reduce the moisture content of the fluoride varnish. The thickness of the applied FV layer was estimated following the same approach as in our previous study [33], using the formula m/ (Aρ). Here, A is the enamel surface area of each specimen (2 × 6 mm^2^), ρ is the FV density (0.006 g/mm^3^, manufacturer data), and m is the applied varnish mass (~0.1 g). Based on these parameters, the theoretical thickness was approximately 1.38 mm. This value represents an estimated average layer thickness calculated from manufacturer specifications; the actual layer may vary due to spreading and micro brush application.

### 2.4. Cold Atmospheric Plasma Setup

Cold atmospheric helium plasma was generated using a dielectric barrier discharge jet (He-DBDJ) custom-built at Amirkabir University of Technology, as previously reported [33]. Helium gas was supplied at a flow rate of 7 L/min through a glass cylinder. A copper ring electrode mounted on one end of the cylinder was driven by a high-voltage source (Vpp = 8 kV, ν = 50 kHz). Due to the dielectric barrier and gas flux, only micro-discharges were formed, producing plasma at near-physiological temperatures (~37 °C). The specimen surface served as the floating counter-electrode. The nozzle-to-surface distance was maintained at 10 mm. Plasma exposure was applied uniformly for 120 s in each treated group (P, PV, VP, and PVP).

### 2.5. Scanning Electron Microscopy (SEM)

The morphological changes in the enamel surface of three specimens from each group were examined using a scanning electron microscope. SEM imaging was performed 24 h after treatment, according to each group’s protocol. Following targeted therapy, the polished and specified areas on the buccal surface (prepared as described in the Specimen Preparation section) were allowed to air dry at room temperature. To enhance the electrical conductivity, a 10 nm gold coating layer was applied to the enamel surfaces of the teeth. Conductivity is attributed to the cleanliness and dryness of a specimen surface. SEM images (FEI Nova, NanoSEM, 450, Thermo Fisher Scientific, Hillsboro, OR, USA) were captured in low vacuum mode with the following parameters: 20 kV, Charge Reduction mode, 18 mm working distance, magnifications of ×1000, ×2000, and ×4000, and aperture ranges of 30, 50, and 100 mm.

### 2.6. Energy-Dispersive X-Ray (EDX)

In this research, we utilized energy-dispersive X-ray Spectroscopy (EDX) to explore the dispersion and distribution of chemical elements on the surface of tooth enamel following the application of plasma and fluoride varnish. We set the EDX parameters to 20-kV energy acceleration voltage and the magnification of 129 times (BRUKER, XFlash 6, 10, Billerica, MA, USA).

Reflected X-rays, systematically scanned the polished treatment enamel surface prepared as described in the specimen preparation section to measure the elements’ atomic percentages. The EDX detector analyzed 700 to 1500 counts per second at three points within the 20 nm range. Calcium, phosphorus, fluoride, carbon, and oxygen were quantified in the tooth enamel at three time points—immediately following therapy, 24 h later, and 48 h later at the surface layer. The Ca/P ratio was calculated as the direct quotient of atomic percentages of calcium and phosphorus obtained from EDX analysis. This ratio was calculated to indicate the enamel’s resistance to decay [34,35].

### 2.7. Microhardness

Following the treatment procedure, the hardness of 10 polished enamel surface (see Specimen Preparation) specimens from each group was measured using a Knoop indenter. The diamond pyramid indenter for the microhardness Vickers test (V-Test II, Bareiss, Burgwald, Germany) with the microhardness angle of 136° between opposite faces was utilized. Bovine enamel was subjected to a 50 g load for 8 s at room temperature, preventing cracking during testing.

Prior to microhardness indentation, specimens were gently blotted with a fibreless tissue and exposed to a stream of room-temperature air to remove only the superficial water film. This step was performed to prevent interference of a liquid layer with indenter contact. Importantly, specimens were otherwise maintained in distilled water storage between measurements to avoid dehydration. Measurements were taken at three points on the specimens’ surface, approximately in the centre. Then, the average was calculated. Three distinct time intervals were used to measure the hardness of the specimens—immediately, 24 h, and 48 h after treatment, to intercept changes in hardness as time progressed. The following equation for the Vickers hardness number (*VHN*) was used:

(1) $$VHN=\frac{2F\sin\frac{136}{2}}{d^{2}}\approx \frac{1.854F}{d^{2}},$$

where the load is represented by *F*, while the indentation diagonal is marked as *d* [27,36].

### 2.8. Statistical Analysis

We have carried out a quantitative analysis of descriptive statistics using the SPSS software (version 29, IBM, Tokyo, Japan). Furthermore, using repeated measure analysis of variance (ANOVA) and Bonferroni tests, we have compared the surface microhardness values, Ca/P ratio, and atomic percentages of fluoride, carbon, and oxygen within the seven groups immediately after, 24 h, and 48 h after the treatment. A significance level of 0.05 was used for all analyses. SEM observations were descriptive only and were not included in statistical analyses.

## 3. Results

### 3.1. SEM Analysis

**Figure 1:**
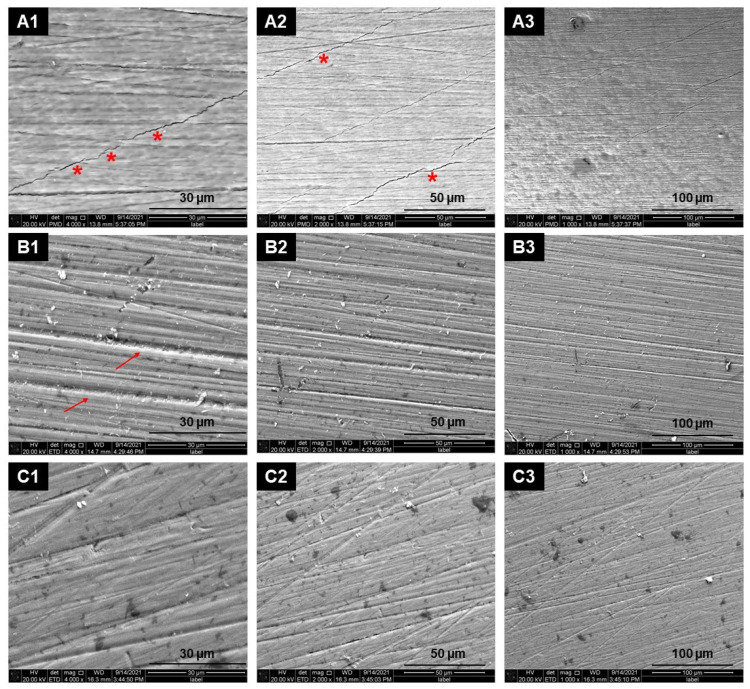
Scanning Electron Microscopy images of the outer buccal enamel surface characteristics after initial polishing. Columns correspond to magnifications of 4000× (1), 2000× (2), and 1000× (3). Rows correspond to the following: (**A**) the control group (**A1**–**A3**), (**B**) Helium gas treatment group (**B1**–**B3**); and (**C**) Helium plasma treatment group (**C1**–**C3**). Cracks and irregularities on the enamel surface marked as (→), uneven pores and holes marked as (*).

In Figure 1, images show the surface characteristics at magnifications of 4000×, 2000×, and 1000× following initial enamel polishing for the control group (A1, A2, and A3), Helium (He) gas treatment (B1, B2, and B3), and Helium (P) plasma treatment (C1, C2, and C3).

In the control group, the polished enamel surface exhibits relative smoothness and uniformity. Additionally, perikymata lines representing horizontal enamel growth are visible.

In the He group, the gas-treated specimens have induced more cracks and irregularities on the enamel surface marked as (→), resulting in uneven pores and holes marked as (*). Despite this, the fundamental structure of tooth enamel remains unaltered. The P group’s irregular holes and pits, similar to those observed in the He group, can be observed on the enamel surface; however, less pronounced.

**Figure 2:**
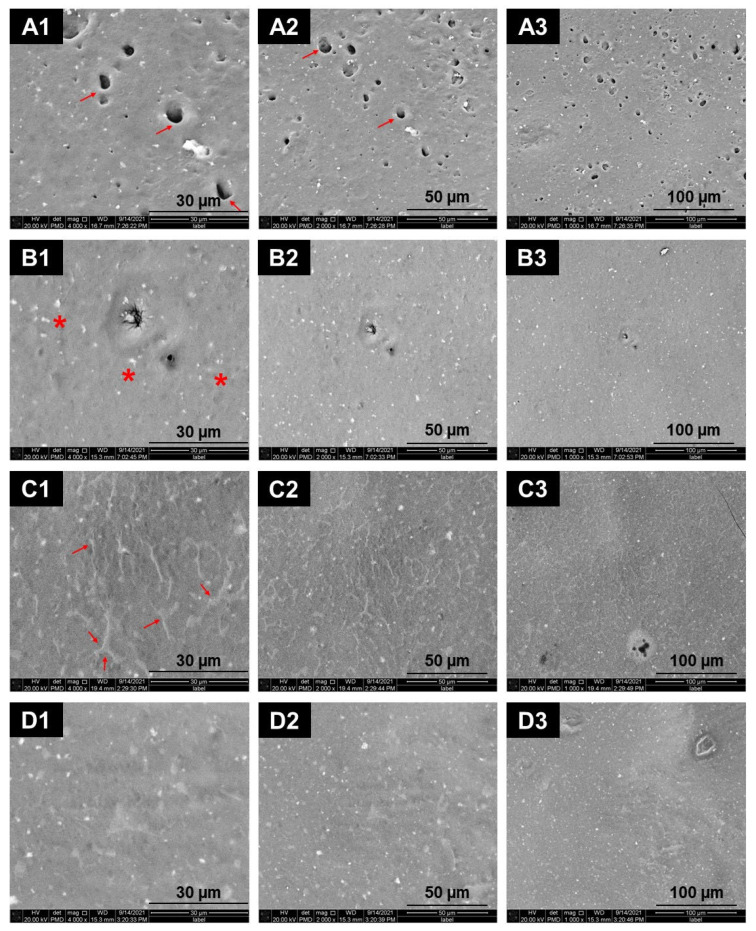
Scanning Electron Microscopy images of the enamel surface morphology after fluoride varnish application (group V) and plasma treatment (groups VP, PV, and PVP). The images depict the outer buccal enamel surface morphology, including areas with residual FV coverage and surface modifications following treatment. Columns correspond to magnifications of 4000× (1), 2000× (2), and 1000× (3). Rows correspond to the following: fluoride varnish (V) group (**A1**–**A3**), fluoride varnish with helium plasma (VP) group (**B1**–**B3**), the helium plasma with following fluoride varnish (PV) group (**C1**–**C3**), and the helium plasma with following varnish and repeated helium plasma therapy (PVP) group (**D1**–**D3**). Formation of holes (marked with →) as a result of fluoride dissolving. The calcium fluoride formations were observed as white spherical regions (marker with *).

The surface morphology of enamel after the fluoride varnish application and plasma therapy is presented in Figure 2 for the following: the fluoride varnish (V) group (A1, A2, and A3), fluoride varnish followed by helium plasma (VP) group (B1, B2, and B3), the helium plasma followed by fluoride varnish (PV) group (C1, C2, and C3), and the helium plasma with following before and after fluoride varnish (PVP) group (D1, D2, and D3).

Figure 2(A1–A3) shows a non-uniform surface coverage, consistent with a relatively thick and uneven fluoride varnish coating on the enamel surface. The image mainly reflects the varnish layer rather than the underlying enamel. The holes (→) within the varnish layer may represent areas of incomplete or irregular coverage. The groups treated with plasma and fluoride varnish showed fewer and smaller instances of such regions. The enamel surface also exhibits fine granulate precipitates with globular structures. White spherical regions (*) are probably derived from the fluoride varnish.

The expression of spherical cells, which correlates with the concentration of calcium fluoride, has increased in the VP group (Figure 2(B1–B3)) as compared to the fluoride varnish group (Figure 2(A1–A3)). Dissolution holes, in contrast to the fluoride varnish group, are less frequent and smaller in diameter. The treated surface displays a more uniform distribution of fluoride. Furthermore, it appears that within this particular group, the quantity of white dots, which indicates the presence of calcium fluoride, exceeds that observed in the V (Figure 2(A1–A3)) and PV (Figure 2(C1–C3)) groups. The PV group (Figure 2(C1–C3)) shows a more consistent spatial distribution of the fluoride varnish on the surface. The surface of the tooth enamel shows no signs of residual fluoride varnish dissolving cavities. Furthermore, all magnifications detect irregular crystalline aggregates. The PVP group (Figure 2(D1–D3)) exhibited the most extensive surface coverage by varnish among all groups. No evidence of erosion or disintegration pits was observed on the enamel surface.

### 3.2. EDX Analysis

Table 2 displays the outcomes of the chemical analysis of bovine enamel using energy-dispersive X-ray spectroscopy (EDX). The constituents of enamel primarily consist of oxygen (O), carbon (C), calcium (Ca), phosphorus (P), and fluorine (F). The following data are summarized in Table 2. Over three time intervals, the EDX data of the enamel surface layer are presented as atomic percentages (at%). The results demonstrate the substantial impact of plasma therapy. The calcium-to-phosphorus ratio (Ca/P) on the initial day was 1.64 for the control group. The helium gas group yielded the same ratio, but the helium plasma group resulted in a ratio of 1.69. Thus, we have not observed any substantial alterations. The calcium-to-phosphorus ratio (Ca/P) on the first day for the FV group was 1.98. The ratio for the groups that received plasma fluoride varnish in addition to VP, PV, and PVP was 2.02, 2.13, and 2.45, respectively (RM-ANOVA, Bonferroni-adjusted *p* < 0.05 compared to control). Over the course of three consecutive days, the Ca/P ratio declined in all groups over time. The PVP group had the highest Ca/P ratio (2.39) after 48 h (*p* < 0.05 vs. all other groups). After two days, the ratio for the group that exclusively received fluoride varnish decreased to 1.95, which was nearly identical to the ratio of 1.97 for the group that initially received coating and then plasma.

Fluoride concentration increased significantly in plasma-treated groups (RM-ANOVA, Bonferroni-adjusted *p* < 0.05), with PVP showing the highest values at 48 h. The control group, which did not receive fluoride varnish, had a negligible amount of fluoride. The fluoride concentration in the control groups, consisting of helium gas and helium plasma, on day zero was 0.03, 0.05, and 0.06, respectively. As anticipated, there is no observable alteration over a period of 48 h. The fluoride concentration after 48 h was highest in the PVP group (7.13%), followed by PV (4.79%), VP (3.33%), and V (1.21%).

The carbon atomic percentage was highest in the control and helium gas groups and lowest in the PVP group after 48 h (*p* < 0.05 compared to control). The carbon levels immediately after the treatment are 43.21 and 42.35 in the control and helium gas groups, respectively. This is the highest value within all groups. After 48 h, the PVP group was at its lowest value of 16.19.

Oxygen content remained lowest and most stable in the control and helium gas groups, while plasma-treated specimens, particularly PV and PVP, exhibited the highest oxygen levels (*p* < 0.05). Throughout the experiments, the levels of oxygen in the control group and the helium gas group were the lowest and most steady. In the PVP and PV groups, the oxygen atomic percentage was the highest.

### 3.3. Microhardness Analysis

Table 3 presents the average Vickers hardness number values of the enamel surface for all groups on the three days of the experiment. On the zeroth day, the measured microhardness was within the range of 323–437 in all groups. After one day, this number was between 311 and 430. On the second day, the number was within the range of 322 to 455. The control group showed marginal changes between days. However, the PVP group demonstrated the most significant change. The remaining groups showed intermediate VHN values between the control and PVP groups across all time points.

The repeated-measures ANOVA was used to evaluate the effects of Group and Time, as well as their interaction, on microhardness levels across three consecutive days. The analysis showed significant main effects of Group and Time, and a significant Group × Time interaction (*p* < 0.05). Therefore, pairwise comparisons were performed using the Bonferroni post hoc test.

All groups, with the exception of those exposed to helium gas flow and helium plasma, had statistically significantly greater microhardness than the control group. The PVP group exhibited the highest VHN at all time points, with a significant difference compared to all other groups (*p* < 0.001).

Over time, the microhardness of the PVP and PV groups exhibited a progressive increase, while the V and VP groups showed more moderate changes. On the second day, the PV group demonstrated significantly higher VHN compared to both VP and V groups (*p* < 0.01).

## 4. Discussion

Multiple studies have highlighted the advantages of fluoride varnish, particularly for young patients, due to its ease of use, safety, and ability to maintain fluoride in saliva while forming calcium fluoride (CaF_2_) on enamel surfaces, serving as a reservoir of fluoride ions. Topical fluoride protects enamel mainly by maintaining fluoride levels through repeated exposure and by forming CaF_2_ deposits that act as temporary reservoirs, releasing ions during acidic challenges [37,38]. In addition to its remineralization effects, fluoride varnish may exert antibacterial and anti-biofilm activity. Fluoride can reduce the acidogenicity of cariogenic bacteria, impair bacterial adhesion, and disrupt biofilm formation [39]. However, this protective effect diminishes over time, requiring repeated applications; therefore, optimizing varnish longevity and effectiveness remains a critical research goal [15,16].

Our pilot data indicated that combining cold atmospheric plasma (CAP) helium with fluoride varnish enhanced fluoride uptake and improved enamel resistance [33]. CAP modifies enamel surfaces through active particles, ions, and radicals generated by electric discharge, increasing surface adhesion, energy, and wettability, which facilitates mineral deposition and fluoride retention [40,41].

These properties make CAP a promising tool for enhancing fluoride varnish performance. Nevertheless, further optimization is needed to identify the ideal combination of fluoride concentration, pH, exposure time, and enamel structure for maximum efficacy [42,43,44]. Additionally, a limitation of this study is that specimens were stored in distilled water, which minimized ionic exchange and microbial growth but does not replicate the oral environment. Therefore, while our in vitro setup allowed controlled isolation of treatment effects, the applicability of the results to real clinical practice remains limited. Saliva dynamics, temperature fluctuations, and microbial biofilms may all alter treatment efficacy. Future ex vivo and clinical studies should therefore validate these findings under physiologic conditions.

SEM is a widely used method for assessing enamel surface morphology and detecting microstructural changes [27,45]. We performed SEM imaging at three magnifications: 1000×, 2000×, and 4000×. The enamel surface evaluated before each treatment process at magnifications ranging from 1000× to 4000× was relatively smooth with few surface defects, which is consistent with Alsabeel’s findings [46]. In the pilot phase [33], we found cracks on the enamel surface, which presented a significant issue. Due to very limited data, we cautiously attributed the appearance of cracks to plasma processing. This was initially attributed to either the momentum transfer of plasma particles or the interaction of local electric fields with the enamel surface. However, the cracks were not visible in the control specimen. Now, by increasing the number of specimens and repeating the SEM scan, we can clearly see cracks and horizontal lines of perikymata in the control specimens, helium gas, and helium plasma. The presence of cracks in nearly all specimens ruled out plasma as the sole cause, contradicting our initial hypothesis in [33]. It should also be noted that some microcracks observed in SEM images may be artefacts from specimen drying and sputter-coating; previous studies have reported similar effects [47].

Bovine enamel grows faster and therefore exhibits larger crystal grains, more defects, and a rougher surface morphology than human enamel [48]. At higher magnifications (2000×, 4000×), helium gas produced deeper cracks than helium plasma. Data from [49] also indicate that water immersion, in addition to thermal changes, contributes to enamel degradation. Plasma-related thermal effects may cause cracking or melting of the enamel’s hexagonal structure. However, no evidence of such thermal damage was observed in helium plasma-treated samples. Next, we examined the SEM images of specimens treated only with fluoride varnish (without plasma exposure). After application, the enamel surface showed a virtually uniform mineral deposit layer, suggesting the formation of a new protective barrier. Prior to varnish, plasma-treated enamel appeared more receptive to mineralization. These findings were consistent with hardness testing. The PV group had a higher average tooth enamel hardness than the VP group. A limitation of our study is that surface roughness was only qualitatively assessed via SEM. Quantitative methods such as atomic force microscopy (AFM) or profilometry should be included in future studies to complement the morphological findings.

The thickness and stickiness of the fluoride varnish layer on treated surfaces changed depending on whether the varnish was applied before or after plasma treatment, as shown by SEM research. Greater cohesiveness and CaF_2_ deposits built a barrier against acid attack, with the PVP group showing the strongest results. The PVP group had a smoother, more cohesive surface with less breakdown than the groups that received fluoride varnish before (VP) or after (PV) the plasma treatment. This likely reflects improved fluoride deposition after plasma, enhancing remineralization. Similar findings have been reported with other remineralizing agents, such as bioglass, where plasma treatment before and after application enhanced cohesion and hardness [36].

Surface morphology cannot provide information on the mechanical qualities, such as hardness and strength, of dental tissues. Surface enamel hardness measurement is a highly sensitive and repeatable approach for investigating the early phases of enamel degradation. However, surface enamel hardness measurements cannot provide information about hardness changes at different enamel levels. Polishing, though potentially altering enamel, was necessary for standardization.

The mechanical properties of healthy bovine enamel, such as microhardness and fracture resistance, have received limited research. However, the literature contains a number of studies that investigate alterations in bovine enamel after exposure to various items, such as bleaching chemicals or acidic solutions [50,51]. For example, Fernández et al. [52] measured hardness at the enamel edge, unlike our buccal surface measurements, which may explain differences in results. Arango-Santander et al. [53] studied mechanical and chemical structural changes in bovine enamel and found similar surface enamel microhardness values to ours. Zhao et al. [54] looked at changes in the microhardness of the surface of bovine enamel before and after being exposed to cold plasma. They found that the enamel was more resistant to breaking down, which supported our findings. Since our study focused solely on surface hardness, comparisons to studies reporting subsurface changes remain limited.

Although no significant difference was seen between V and PVP on day 1, all plasma–varnish combinations increased microhardness compared to control and helium gas. The PVP group showed the greatest improvement, while PV also outperformed V after 48 h. Plasma thus enhanced enamel properties without morphological damage (SEM). Importantly, plasma also improved varnish adhesion, making the layer more durable. The enhanced effect is likely due to CaF_2_ layer formation, which serves as both a mineral reservoir and protective barrier.

In this work, we used energy-dispersive X-ray Analysis (EDX) to determine the types of elements present on the enamel surface and their percentages in the enamel structure. Our previous study [33] relied on ion-selective electrodes, which cannot directly assess enamel surface fluoride. In contrast, EDX enables the accurate identification of surface elements in atomic percentages [55,56]. As a result, our research group did this investigation in three stages, beginning immediately after the experiment and continuing for up to 48 h. Nonetheless, the current EDX evaluation showed no significant damage to the enamel surface, indicating enamel resilience, which correlates with calcium and phosphate levels and may be common elements in other tests. Enamel hydroxyapatite undergoes ionic substitutions (e.g., carbon, fluoride), which influence solubility and decay resistance. Areas low in calcium and high in carbonate are particularly vulnerable to decay, but substituting fluoride for hydroxyl may improve decay resistance.

In the investigation, all specimens without fluoride varnish, including the control group, helium gas, and helium plasma, had less than 0.06 percent fluoride, which was consistent with previous group studies [57]. Fluoride varnish with plasma markedly increased fluoride retention over time. All plasma and fluoride varnish groups showed significant increases in fluoride levels at all three time points when compared to the fluoride varnish group. Prolonged fluoride exposure promotes fluorapatite formation, enhancing enamel resistance [58]. The Ca/P ratio is widely used as an indicator of mineralization. In the literature, the Ca/P ratio of bovine enamel has been reported in the range of approximately 1.5–1.7, depending on the analytical method and specimen source [27,56]. Ratios closer to 1.67 reflect stoichiometric hydroxyapatite, whereas lower values indicate demineralization, and higher values (>2.1) may reflect relative calcium enrichment or reduced phosphate content [59,60]. The control group had a Ca/P ratio of 1.64 to 1.62 after 48 h, which was similar to findings from prior investigations [57]. These findings align with studies linking higher Ca and P to improved hardness [27]. In contrast, the PV, VP, and especially the PVP groups showed increased Ca/P values, reaching up to 2.39 after 48 h. This increase is in line with prior studies reporting that plasma-assisted fluoride treatments promote mineralization and shift the Ca/P ratio toward values associated with improved enamel strength and resistance [60]. Plasma treatment further enhanced surface properties by promoting ion exchange (e.g., Na^+^ with H^+^ or H_3_O^+^) and creating a supersaturated environment favourable for apatite formation [57].

Furthermore, higher carbonate levels are associated with lower crystallinity and hardness, indicating that carbonate is a critical component of tooth enamel, influencing its mechanical qualities. Carbon may originate from enamel organics or surface contamination [61]. Consistent with this trend, the EDX data suggested relatively lower carbon signals in the PVP group and higher in the control and helium gas groups. While this pattern may reflect differences in carbon content, we acknowledge that EDX cannot distinguish between carbonate and other carbon-containing species; therefore, our interpretation is limited to relative carbon content rather than definitive carbonate levels. Furthermore, the small number of specimens analyzed (*n* = 3 per group) limits the strength of this conclusion. Additional studies with larger specimen sizes and complementary techniques (e.g., FTIR, Raman spectroscopy) would be needed to confirm changes in carbonate content.

## 5. Conclusions

In this in vitro study, the sequential application of cold helium plasma before and after fluoride varnish (PVP) resulted in the highest increase in surface fluoride atomic percentage (from 1.21% to 7.31% at 48 h) and a statistically significant increase in surface microhardness (an increase of approximately 24%; *p* < 0.001) compared to varnish alone. These findings suggest that the sequence of CAP application affects varnish performance. Further in vivo and longer-term studies are required to confirm clinical relevance and to optimize CAP-fluoride protocols.

## Figures and Tables

**Table 1:** Experimental groups and their treatment.

Group	Abbreviation	Treatment
Control	C	Polished + No treatment
Helium gas	He	Polished + Helium Gas
Helium Plasma	P	Polished + Helium Plasma Treatment
Fluoride Varnish	V	Polished + Fluoride Varnish
Plasma → Varnish	PV	Polished + Helium Plasma Treatment + Fluoride Varnish
Varnish → Plasma	VP	Polished + Fluoride Varnish + Helium Plasma Treatment
Plasma → Varnish → Plasma	PVP	Polished + Helium Plasma Treatment + Fluoride Varnish + Helium Plasma Treatment

**Table 2:** Effects of plasma and varnish enamel treatments within the groups. The chemical compounds’ atomic percentages measured using the energy-dispersive X-ray spectroscopy (EDX).

				Element			
Group	Day	Ca (at%)	P (at%)	Ca/P	F (at%)	C (at%)	O (at%)
Control	Day_0	21.48	13.04	1.64	0.03	43.21	21.78
	Day_1	21.54	13.12	1.63	0.02	42.41	21.29
	Day_2	21.59	13.20	1.62	0.02	42.15	21.20
He-Gas	Day_0	20.96	13.14	1.59	0.05	42.35	22.58
	Day_1	20.94	13.17	1.58	0.03	42.15	22.13
	Day_2	21.03	13.31	1.57	0.02	42.12	21.82
He-Plasma	Day_0	21.77	12.85	1.69	0.06	25.60	36.03
	Day_1	21.54	12.65	1.70	0.05	24.81	35.95
	Day_2	21.60	12.77	1.68	0.06	24.42	35.82
Varnish	Day_0	23.36	11.76	1.98	2.29	33.11	28.37
	Day_1	22.96	11.67	1.96	1.85	34.55	27.71
	Day_2	22.94	11.68	1.95	1.21	34.00	27.35
Plasma-Varnish	Day_0	24.95	11.45	2.17	5.79	18.67	38.10
	Day_1	23.88	11.53	2.13	5.24	18.68	37.78
	Day_2	23.78	11.60	2.13	4.79	18.38	37.36
Varnish-Plasma	Day_0	24.49	12.06	2.02	4.39	19.19	37.35
	Day_1	23.88	12.09	1.98	3.80	18.43	36.86
	Day_2	23.78	12.02	1.97	3.33	18.17	36.47
Plasma-Varnish-Plasma	Day_0	24.78	10.08	2.45	8.09	17.79	38.34
	Day_1	24.65	10.26	2.39	7.69	16.62	38.01
	Day_2	24.73	10.32	2.39	7.31	16.19	37.86

**Table 3:** Mean values of the Vickers hardness number at three consecutive days of the experiment for all groups. Values within the same column/row that share a common superscript letter are not significantly different (*p* < 0.05, Bonferroni post hoc test). Different letters indicate statistically significant differences.

	Time	Day_0	Day_1	Day_2
Groups		Mean ± SD	Mean ± SD	Mean ± SD
Control		317.50 ± 47.84 ^a^	316.46 ± 32.83 ^a^	316.90 ± 25.51 ^a^
He-gas		336.73 ± 34.08 ^a^	319.60 ± 35.23 ^a^	304.96 ± 32.64 ^a^
He-plasma		402.20 ± 43.11 ^b^	370.23 ± 65.12 ^b^	359.50 ± 55.21 ^b^
Varnish		400.90 ± 115.10 ^b^	370.76 ± 75.33 ^b^	351.36 ± 71.27 ^b^
Plasma-Varnish		419.36 ± 28.63 ^bc^	396.53 ± 21.58 ^bc^	381.13 ± 23.41 ^bc^
Varnish-Plasma		398.56 ± 39.25 ^b^	369.23 ± 37.49 ^b^	382.53 ± 47.71 ^bc^
Plasma-Varnish-Plasma		437.43 ± 58.03 ^c^	430.57 ± 32.35 ^c^	455.87 ± 44.62 ^c^

## Data Availability

The original contributions presented in this study are included in the article and/or Supplementary Materials. Further inquiries can be directed to the corresponding author.

## Supplementary Materials

The following supporting information can be downloaded at: https://www.mdpi.com/article/10.3390/ma18194466/s1.

## References

[1] Karpiński T.M., Szkaradkiewicz A.K. (2013). Microbiology of dental caries. J. Biol. Earth Sci..

[2] Santonocito S., Polizzi A., Isola G. (2025). The Impact of Diet and Nutrition on the Oral Microbiome. Oral Microbiome: Symbiosis, Dysbiosis and Microbiome Interventions for Maintaining Oral and Systemic Health.

[3] Nath S., Zilm P., Jamieson L., Santiago P.H.R., Ketagoda D.H.K., Weyrich L. (2025). The influence of diet, saliva, and dental history on the oral microbiome in healthy, caries-free Australian adults. Sci. Rep..

[4] Yadav K., Prakash S. (2017). Dental caries: A microbiological approach. J. Clin. Infect. Dis. Pract..

[5] Naim S., Spagnuolo G., Osman E., Mahdi S.S., Battineni G., Qasim S.S.B., Cernera M., Rifai H., Jaafar N., Maalouf E. (2022). Quantitative measurements of the depth of enamel demineralization before and after bleach: An in vitro study. BioMed Res. Int..

[6] Whelton H., Spencer A., Do L., Rugg-Gunn A. (2019). Fluoride revolution and dental caries: Evolution of policies for global use. J. Dent. Res..

[7] Kitasako Y., Sadr A., Hamba H., Ikeda M., Tagami J. (2012). Gum containing calcium fluoride reinforces enamel subsurface lesions in situ. J. Dent. Res..

[8] Hariri I., Sadr A., Nakashima S., Shimada Y., Tagami J., Sumi Y. (2013). Estimation of the enamel and dentin mineral content from the refractive index. Caries Res..

[9] Featherstone J.D.B. (1999). Prevention and reversal of dental caries: Role of low level fluoride. Community Dent. Oral Epidemiol..

[10] Hamilton I.R. (1990). Biochemical effects of fluoride on oral bacteria. J. Dent. Res..

[11] Wong M.C.M., Zhang R., Luo B.W., Glenny A.-M., Worthington H.V., Lo E.C.M. (2010). Topical fluoride as a cause of dental fluorosis in children. Cochrane Database Syst. Rev..

[12] Carvalho D.M., Salazar M., Oliveira B.H.d., Coutinho E.S.F. (2010). Fluoride varnishes and decrease in caries incidence in preschool children: A systematic review. Rev. Bras. Epidemiol..

[13] Gao S.S., Zhang S., Mei M.L., Lo E.C.-M., Chu C.-H. (2016). Caries remineralisation and arresting effect in children by professionally applied fluoride treatment–a systematic review. BMC Oral Health.

[14] Chou R., Cantor A., Zakher B., Mitchell J.P., Pappas M. (2013). Preventing dental caries in children< 5 years: Systematic review updating USPSTF recommendation. Pediatrics.

[15] Kalnina J., Care R. (2016). Prevention of occlusal caries using a ozone, sealant and fluoride varnish in children. Stomatologija.

[16] Azevedo D.T., Faraoni-Romano J.J., Derceli J.d.R., Palma-Dibb R.G. (2012). Effect of Nd: YAG laser combined with fluoride on the prevention of primary tooth enamel demineralization. Braz. Dent. J..

[17] Tabares F.L., Junkar I. (2021). Cold plasma systems and their application in surface treatments for medicine. Molecules.

[18] Ghadirian F., Abbasi H., Bavi O., Naeimabadi A. (2023). How living cells are affected during the cold atmospheric pressure plasma treatment. Free Radic. Biol. Med..

[19] Soltani Z., Mehrabifard R., Rezaie F., Hatami M.M., Soltani H. (2024). Simulation of the impact of humidity on the species generated by a one-dimensional discharge of helium gas. arXiv.

[20] Fathollah S., Mirpour S., Mansouri P., Dehpour A.R., Ghoranneviss M., Rahimi N., Safaie Naraghi Z., Chalangari R., Chalangari K.M. (2016). Investigation on the effects of the atmospheric pressure plasma on wound healing in diabetic rats. Sci. Rep..

[21] Reuter S., Von Woedtke T., Weltmann K.-D. (2018). The kINPen—A review on physics and chemistry of the atmospheric pressure plasma jet and its applications. J. Phys. D Appl. Phys..

[22] Mirpour S., Fathollah S., Mansouri P., Larijani B., Ghoranneviss M., Mohajeri Tehrani M., Amini M.R. (2020). Cold atmospheric plasma as an effective method to treat diabetic foot ulcers: A randomized clinical trial. Sci. Rep..

[23] Shojaei E., Zare S., Shirkavand A., Eslami E., Fathollah S., Mansouri P. (2022). Biophysical evaluation of treating adipose tissue-derived stem cells using non-thermal atmospheric pressure plasma. Sci. Rep..

[24] Mehrabifard R. (2023). Investigating the Effects of Cold Plasma on Cancer Cell Migration in the Presence of a Static Magnetic Field. Meet. Abstr..

[25] Gershater E., Griswold O., Talsania B.E., Zhang Y., Chung C.-H., Zheng Z., Li C. (2023). Effects of plasma treatment on the strength of bonding to ceramic surfaces in orthodontics—A comprehensive review. Bioengineering.

[26] Santonocito S., Ferlito S., Polizzi A., Ronsivalle V., Sclafani R., Valletta A., Lo Giudice A., Cavalcanti R., Spagnuolo G., Isola G. (2022). Therapeutic and metagenomic potential of the biomolecular therapies against periodontitis and the oral microbiome: Current evidence and future perspectives. Int. J. Mol. Sci..

[27] El-Wassefy N.A. (2017). Remineralizing effect of cold plasma and/or bioglass on demineralized enamel. Dent. Mater. J..

[28] Rupf S., Lehmann A., Hannig M., Schäfer B., Schubert A., Feldmann U., Schindler A. (2010). Killing of adherent oral microbes by a non-thermal atmospheric plasma jet. J. Med. Microbiol..

[29] Duske K., Koban I., Kindel E., Schröder K., Nebe B., Holtfreter B., Jablonowski L., Weltmann K.D., Kocher T. (2012). Atmospheric plasma enhances wettability and cell spreading on dental implant metals. J. Clin. Periodontol..

[30] Valizadeh S., Farhadi E., Moradi A., Hashemikamangar S.S. (2021). Evaluation of the Effect of Cold Plasma Treatment on the Microshear Bond Strength of Composite Resin Restorations to Dentin using Different Adhesive Systems and the Effect of Thermocycling. Open Dent. J..

[31] Kim Y.M., Lee H.Y., Lee H.J., Kim J.B., Kim S., Joo J.Y., Kim G.C. (2018). Retention improvement in fluoride application with cold atmospheric plasma. J. Dent. Res..

[32] Bapat S.A., Shashikiran N.D., Gugawad S., Gaonkar N., Taur S., Hadakar S., Chaudhari P. (2022). Effect of non-thermal atmospheric pressure plasma and ErCr: YSGG LASER activation of three fluoride varnishes on surface re-mineralization of enamel: A SEM-EDX analysis. J. Indian Soc. Pedod. Prev. Dent..

[33] Fathollah S., Abbasi H., Akhoundi S., Naeimabadi A., Emamjome S. (2022). Cold plasma enamel surface treatment to increase fluoride varnish uptake. Sci. Rep..

[34] Koontongkaew S., Utispan K., Chawhuaveang D.D., Yu O.Y., Worawongvasu R. (2024). Enamel and Its Interaction with the Oral Environment. Enamel and Dentin-Pulp Complex.

[35] Cîrdei M.-V., Margan M.-M., Margan R., Ban-Cucerzan A., Petre I., Hulka I., Horhat R.M., Todea D.C. (2024). Surface and Mineral Changes of Primary Enamel after Laser Diode Irradiation and Application of Remineralization Agents: A Comparative In Vitro Study. Children.

[36] El-Wassefy N.A. (2017). The effect of plasma treatment and bioglass paste on enamel white spot lesions. Saudi J. Dent. Res..

[37] Petersen P.E., Bourgeois D., Ogawa H., Estupinan-Day S., Ndiaye C. (2005). The global burden of oral diseases and risks to oral health. Bull. World Health Organ..

[38] Oliveira M.R.C., Oliveira P.H.C., Oliveira L.H.C., Horliana A.C.R.T., Cesar P.F., Moura S.K., Bussadori S.K. (2019). Microhardness of bovine enamel after different fluoride application protocols. Dent. Mater. J..

[39] Matar M., Darwish S., Salma R., Lotfy W. (2023). Evaluation of the antibacterial activity of Enamelast^®^ and Fluor defender^®^ fluoride varnishes against Streptococcus mutans biofilm: An in vitro study in primary teeth. Eur. Arch. Paediatr. Dent..

[40] Ritts A.C., Li H., Yu Q., Xu C., Yao X., Hong L., Wang Y. (2010). Dentin surface treatment using a non-thermal argon plasma brush for interfacial bonding improvement in composite restoration. Eur. J. Oral Sci..

[41] Teixeira H.S., Coelho P.G., Duarte S., Janal M.N., Silva N., Thompson V.P. (2015). Influence of atmospheric pressure plasma treatment on mechanical proprieties of enamel and sealant bond strength. J. Biomed. Mater. Res. Part B Appl. Biomater..

[42] Tenuta L.M.A., Zamataro C.B., Del Bel Cury A.A., Tabchoury C.P.M., Cury J.A. (2009). Mechanism of fluoride dentifrice effect on enamel demineralization. Caries Res..

[43] Richards D. (2015). Fluoride gel effective at reducing caries in children. Evid.-Based Dent..

[44] Hafith A.N., Zbidi N.D., Hasan S.M., Shallal W. (2024). Research on Treating Demineralized Enamel with Different Remineralizing Agents before Bonding Orthodontic Brackets. Metall. Mater. Eng..

[45] Souza-Gabriel A.E., Colucci V., Turssi C.P., Serra M.C., Corona S.A.M. (2010). Microhardness and SEM after CO2 laser irradiation or fluoride treatment in human and bovine enamel. Microsc. Res. Tech..

[46] Alsabeel M.H., Qasim A.A. (2024). Impact of Fluoridated Dental Products on Surface Roughness and Morphology of Bleached Tooth Enamel: An In Vitro Study. Pharmacogn. J..

[47] Rödig T., Dullin C., Kück F., Krebs M., Hettwer-Steeger I., Haupt F. (2022). Influence of moisture content of frozen and embalmed human cadavers for identification of dentinal microcracks using micro-computed tomography. J. Mech. Behav. Biomed. Mater..

[48] Nakamichi I., Iwaku M., Fusayama T. (1983). Bovine teeth as possible substitutes in the adhesion test. J. Dent. Res..

[49] Taube F., Ylmén R., Shchukarev A., Nietzsche S., Norén J.G. (2010). Morphological and chemical characterization of tooth enamel exposed to alkaline agents. J. Dent..

[50] Khoubrouypak Z., Abbasi M., Ahmadi E., Rafeie N., Behroozibakhsh M. (2021). Effect of Cold Atmospheric Pressure Plasma Coupled with Resin-Containing and Xylitol-Containing Fluoride Varnishes on Enamel Erosion. Int. J. Dent..

[51] Zanet C.G., Fava M., Alves L.A.C. (2011). In Vitro evaluation of the microhardness of bovine enamel exposed to acid solutions after bleaching. Braz. Oral Res..

[52] Fernández T E., Abbiati C N., Cabrera A J., Martínez M R. (2011). Dental enamel micro-hardness for permanent central incisors in two beef cattle genotypes. Rev. MVZ Córdoba.

[53] Arango-Santander S., Montoya C., Pelaez-Vargas A., Ossa E.A. (2020). Chemical, structural and mechanical characterization of bovine enamel. Arch. Oral Biol..

[54] Zhao H., Wang X., Liu Z., Wang Y., Zou L., Chen Y., Han Q. (2023). The effect of argon cold atmospheric plasma on the metabolism and demineralization of oral plaque biofilms. Front. Cell. Infect. Microbiol..

[55] Möhring S., Cieplik F., Hiller K.-A., Ebensberger H., Ferstl G., Hermens J., Zaparty M., Witzgall R., Mansfeld U., Buchalla W. (2023). Elemental compositions of enamel or dentin in human and bovine teeth differ from murine teeth. Materials.

[56] Scholz K.J., Federlin M., Hiller K.-A., Ebensberger H., Ferstl G., Buchalla W. (2019). EDX-analysis of fluoride precipitation on human enamel. Sci. Rep..

[57] Viana P.S., Orlandi M.O., Pavarina A.C., Machado A.L., Vergani C.E. (2018). Chemical composition and morphology study of bovine enamel submitted to different sterilization methods. Clin. Oral Investig..

[58] Park S.-A., Son J., Kim A.-J., Oh S., Bae J.-M. (2024). Effect of adhesive components in experimental fluoride varnish on fluoride release within 30 days in vitro study. Dent. Mater. J..

[59] Lussi A., Bossen A., Höschele C., Beyeler B., Megert B., Meier C., Rakhmatullina E. (2012). Effects of enamel abrasion, salivary pellicle, and measurement angle on the optical assessment of dental erosion. J. Biomed. Opt..

[60] Arnold W., Gaengler P. (2007). Quantitative analysis of the calcium and phosphorus content of developing and permanent human teeth. Ann. Anat.-Anat. Anz..

[61] Mine A., Yoshida Y., Suzuki K., Nakayama Y., Yatani H., Kuboki T. (2006). Spectroscopic characterization of enamel surfaces irradiated with Er: YAG laser. Dent. Mater. J..

