# Supplementary material for: Combination of Cold Helium Plasma with Fluoride Varnish to Improve Enamel Surface Protection
Source: Materials (Basel). 2025 Sep 25;18(19):4466. doi: 10.3390/ma18194466 (PMC12525247; doi:10.3390/ma18194466)
Supplement: Supplementary file 1 [file materials-18-04466-s001.zip › Raw Result/EDX/EDX_Day0.pdf]

EDX_Day_0

Control_1

El	AN	Series	unn. C [wt.%]	norm. C [wt.%]	Atom. C [at.%]	Error (1 Sigma) [wt.%]
C	6	K-series	58.91	58.91	44.25	7.13
O	8	K-series	34.09	34.09	22.78	4.65
N	7	K-series	3.55	3.55	0.17	1.05
Si	14	K-series	1.36	1.36	0.00	0.09
P	15	K-series	1.10	1.10	12.48	0.07
Ca	20	K-series	0.72	0.72	20.24	0.06
F	9	K-series	0.17	0.17	0.02	0.12
Mg	12	K-series	0.05	0.05	0.03	0.03
Na	11	K-series	0.05	0.05	0.03	0.03
		Total:	100.00	100.00	100.00	

Control_2

El	AN	Series	unn. C [wt.%]	norm. C [wt.%]	Atom. C [at.%]	Error (1 Sigma) [wt.%]
C	6	K-series	59.51	59.51	43.81	7.97
O	8	K-series	34.88	34.88	20.40	5.53
N	7	K-series	2.37	2.37	0.17	1.16
Si	14	K-series	1.64	1.64	0.00	0.11
P	15	K-series	0.79	0.79	13.34	0.07
Ca	20	K-series	0.47	0.47	22.16	0.06
F	9	K-series	0.23	0.23	0.07	0.21
Na	11	K-series	0.09	0.09	0.05	0.00
Mg	12	K-series	0.00	0.00	0.00	0.00
		Total:	100.00	100.00	100.00	

Control_3

El	AN	Series	unn. C [wt.%]	norm. C [wt.%]	Atom. C [at.%]	Error (1 Sigma) [wt.%]
C	6	K-series	13.28	53.83	41.57	2.47
O	8	K-series	6.73	27.28	22.18	1.59
N	7	K-series	1.59	6.35	0.71	0.88
P	15	K-series	1.38	5.63	13.30	0.09
Si	14	K-series	0.90	3.69	0.04	0.07
Ca	20	K-series	0.74	2.98	22.06	0.06
F	9	K-series	0.00	0.00	0.00	0.17
Na	11	K-series	0.03	0.14	0.08	0.03
Mg	12	K-series	0.03	0.11	0.06	0.03
		Total:	24.66	100.00	100.00	

Helium gas_1

El	AN	Series	unn. C [wt.%]	norm. C [wt.%]	Atom. C [at.%]	Error (1 Sigma) [wt.%]
C	6	K-series	59.22	59.22	41.99	7.59
O	8	K-series	33.51	33.51	22.45	4.95
N	7	K-series	2.24	2.24	0.37	0.95
Si	14	K-series	1.90	1.90	0.12	0.11
P	15	K-series	1.26	1.26	13.55	0.08
Ca	20	K-series	1.08	1.08	21.37	0.07
F	9	K-series	0.65	0.65	0.07	0.30
Na	11	K-series	0.11	0.11	0.07	0.04
Mg	12	K-series	0.02	0.02	0.01	0.03
		Total:	100.00	100.00	100.00	

Helium gas_2

El	AN	Series	unn. C [wt.%]	norm. C [wt.%]	Atom. C [at.%]	Error (1 Sigma) [wt.%]
C	6	K-series	57.23	57.23	42.85	8.16
O	8	K-series	33.78	33.78	22.74	5.81
N	7	K-series	4.26	4.26	0.64	1.89
Si	14	K-series	1.73	1.73	0.84	0.12
P	15	K-series	1.17	1.17	12.51	0.09
Ca	20	K-series	1.01	1.01	20.34	0.08
F	9	K-series	0.72	0.72	0.02	0.43
Na	11	K-series	0.07	0.07	0.04	0.04
Mg	12	K-series	0.04	0.04	0.02	0.03
		Total:	100.00	100.00	100.00	

Helium gas_3

El	AN	Series	unn. C [wt.%]	norm. C [wt.%]	Atom. C [at.%]	Error (1 Sigma) [wt.%]
C	6	K-series	57.75	57.75	42.21	8.17
O	8	K-series	34.87	34.87	22.56	5.91
N	7	K-series	3.36	3.36	0.57	1.62
Si	14	K-series	1.83	1.83	0.02	0.12
P	15	K-series	0.87	0.87	13.38	0.08
F	9	K-series	0.69	0.69	0.06	0.40
Ca	20	K-series	0.58	0.58	21.19	0.07
Mg	12	K-series	0.05	0.05	0.01	0.03
Na	11	K-series	0.00	0.00	0.00	0.00
		Total:	100.00	100.00	100.00	

El	AN	Series	unn. C [wt.%]	norm. C [wt.%]	Atom. C [at.%]	Error (1 Sigma) [wt.%]
C	6	K-series	59.51	59.51	26.76	7.80
O	8	K-series	34.74	34.74	35.26	5.37
N	7	K-series	2.73	2.73	1.70	1.19
Si	14	K-series	1.28	1.28	0.62	0.09
Ca	20	K-series	0.75	0.75	22.25	0.07
P	15	K-series	0.74	0.74	13.32	0.07
F	9	K-series	0.17	0.17	0.05	0.17
Na	11	K-series	0.04	0.04	0.02	0.03
Mg	12	K-series	0.03	0.03	0.02	0.03
		Total:	100.00	100.00	100.00	

Plasma_1

El	AN	Series	unn. C [wt.%]	norm. C [wt.%]	Atom. C [at.%]	Error (1 Sigma) [wt.%]
C	6	K-series	56.11	56.11	24.38	7.61
O	8	K-series	34.89	34.89	36.06	5.45
N	7	K-series	3.09	3.09	4.04	1.31
Ca	20	K-series	2.26	2.26	21.78	0.11
P	15	K-series	1.78	1.78	12.79	0.11
Si	14	K-series	1.67	1.67	0.82	0.11
Na	11	K-series	0.10	0.10	0.06	0.04
F	9	K-series	0.08	0.08	0.06	0.12
Mg	12	K-series	0.02	0.02	0.01	0.03
		Total:	100.00	100.00	100.00	

Plasma_2

El	AN	Series	unn. C [wt.%]	norm. C [wt.%]	Atom. C [at.%]	Error (1 Sigma) [wt.%]
C	6	K-series	58.18	58.18	25.67	9.47
O	8	K-series	33.98	33.98	36.79	7.06
N	7	K-series	3.84	3.84	3.63	2.47
Si	14	K-series	1.78	1.78	0.06	0.13
P	15	K-series	1.06	1.06	12.46	0.10
Ca	20	K-series	0.88	0.88	21.30	0.10
F	9	K-series	0.29	0.29	0.07	0.36
Na	11	K-series	0.00	0.00	0.00	0.03
Mg	12	K-series	0.01	0.01	0.02	0.00
		Total:	100.00	100.00	100.00	

Plasma_3

El	AN	Series	unn. C [wt.%]	norm. C [wt.%]	Atom. C [at.%]	Error (1 Sigma) [wt.%]
O	8	K-series	18.18	36.30	28.59	3.48
Ca	20	K-series	11.63	23.22	23.62	0.39
C	6	K-series	10.57	21.11	32.21	2.47
P	15	K-series	6.58	13.14	11.78	0.29
N	7	K-series	2.76	5.51	1.20	1.30
Na	11	K-series	0.16	0.32	0.26	0.05
F	9	K-series	0.13	0.26	2.25	0.17
Si	14	K-series	0.06	0.12	0.08	0.03
Mg	12	K-series	0.01	0.02	0.01	0.03
		Total:	50.08	100.00	100.00	

Varnish_1

El	AN	Series	unn. C [wt.%]	norm. C [wt.%]	Atom. C [at.%]	Error (1 Sigma) [wt.%]
O	8	K-series	15.34	29.43	28.56	3.29
Ca	20	K-series	12.56	24.10	22.97	0.43
C	6	K-series	12.49	23.96	33.39	2.90
P	15	K-series	6.98	13.40	11.89	0.31
N	7	K-series	3.84	7.37	0.49	1.75
F	9	K-series	0.72	1.38	2.33	0.43
Mg	12	K-series	0.13	0.24	0.08	0.04
Si	14	K-series	0.06	0.11	0.07	0.03
Na	11	K-series	0.00	0.00	0.22	0.00
		Total:	52.12	100.00	100.00	

Varnish_2

El	AN	Series	unn. C [wt.%]	norm. C [wt.%]	Atom. C [at.%]	Error (1 Sigma) [wt.%]
O	8	K-series	18.52	36.50	27.98	3.68
C	6	K-series	11.45	22.56	33.75	2.72
Ca	20	K-series	10.75	21.18	23.49	0.37
P	15	K-series	6.68	13.16	11.63	0.30
N	7	K-series	3.00	5.91	0.39	1.46
F	9	K-series	0.16	0.31	2.29	0.20
Mg	12	K-series	0.07	0.14	0.10	0.04
Si	14	K-series	0.07	0.13	0.08	0.03
Na	11	K-series	0.06	0.11	0.29	0.04
		Total:	50.73	100.00	100.00	

Varnish_3

El	AN	Series	unn. C [wt.%]	norm. C [wt.%]	Atom. C [at.%]	Error (1 Sigma) [wt.%]
O	8	K-series	30.05	40.08	38.14	5.27
Ca	20	K-series	23.05	30.74	24.66	0.73
P	15	K-series	10.64	14.18	11.35	0.45
C	6	K-series	7.90	10.54	17.91	2.12
N	7	K-series	2.45	3.27	0.78	1.28
F	9	K-series	0.54	0.71	5.77	0.37
Na	11	K-series	0.18	0.24	0.21	0.05
Si	14	K-series	0.11	0.15	0.61	0.04
Mg	12	K-series	0.06	0.09	0.57	0.03
		Total:	74.99	100.00	100.00	

Plasma+Varnish_1

El	AN	Series	unn. C [wt.%]	norm. C [wt.%]	Atom. C [at.%]	Error (1 Sigma) [wt.%]
Ca	20	K-series	26.27	37.58	25.59	0.81
O	8	K-series	21.85	31.25	38.09	3.82
P	15	K-series	11.73	16.78	11.90	0.49
C	6	K-series	7.30	10.44	18.09	1.78
N	7	K-series	2.01	2.88	0.01	0.99
Na	11	K-series	0.40	0.57	0.10	0.07
Mg	12	K-series	0.15	0.22	0.01	0.04
F	9	K-series	0.11	0.16	6.19	0.14
Si	14	K-series	0.08	0.12	0.02	0.03
		Total:	69.92	100.00	100.00	

Plasma+Varnish_2

El	AN	Series	unn. C [wt.%]	norm. C [wt.%]	Atom. C [at.%]	Error (1 Sigma) [wt.%]
Ca	20	K-series	26.67	40.11	24.62	0.82
O	8	K-series	17.93	26.96	38.09	3.08
P	15	K-series	11.95	17.98	11.12	0.50
C	6	K-series	7.08	10.65	18.03	1.58
N	7	K-series	2.04	3.06	0.95	0.89
Al	13	K-series	0.33	0.50	0.42	0.05
F	9	K-series	0.23	0.35	5.42	0.17
Na	11	K-series	0.17	0.25	0.15	0.04
Si	14	K-series	0.06	0.10	0.62	0.03
Mg	12	K-series	0.03	0.04	0.58	0.03
		Total:	66.49	100.00	100.00	

Plasma+Varnish_3

El	AN	Series	unn. C [wt.%]	norm. C [wt.%]	Atom. C [at.%]	Error (1 Sigma) [wt.%]
Ca	20	K-series	27.94	44.90	24.93	0.86
P	15	K-series	13.18	21.18	12.44	0.55
O	8	K-series	10.01	16.30	37.00	2.59
C	6	K-series	5.52	10.48	20.97	1.72
N	7	K-series	2.21	3.55	0.10	1.14
Na	11	K-series	0.17	0.28	0.39	0.05
Si	14	K-series	0.16	0.26	0.10	0.04
Mg	12	K-series	0.04	0.07	0.07	0.03
F	9	K-series	3.00	3.00	4.00	0.00
		Total:	62.22	100.00	100.00	

Varnish+Plasma_1

El	AN	Series	unn. C [wt.%]	norm. C [wt.%]	Atom. C [at.%]	Error (1 Sigma) [wt.%]
Ca	20	K-series	31.57	44.10	23.58	0.97
P	15	K-series	15.73	21.97	11.46	0.65
O	8	K-series	14.78	20.64	37.40	3.12
C	6	K-series	6.47	9.04	18.33	1.77
N	7	K-series	1.85	2.58	3.49	1.09
Al	13	K-series	0.39	0.55	0.50	0.06
F	9	K-series	0.32	0.45	4.58	0.25
Na	11	K-series	0.27	0.38	0.40	0.06
Si	14	K-series	0.15	0.21	0.18	0.04
Mg	12	K-series	0.06	0.08	0.08	0.04
		Total:	71.59	100.00	100.00	

Varnish+Plasma_2

El	AN	Series	unn. C [wt.%]	norm. C [wt.%]	Atom. C [at.%]	Error (1 Sigma) [wt.%]
Ca	20	K-series	31.59	44.42	24.98	0.97
P	15	K-series	15.66	22.02	12.30	0.65
O	8	K-series	14.81	20.82	37.67	3.12
C	6	K-series	6.41	9.02	18.28	1.75
N	7	K-series	1.85	2.60	1.52	1.09
F	9	K-series	0.33	0.46	4.59	0.25
Na	11	K-series	0.27	0.38	0.41	0.06
Si	14	K-series	0.14	0.20	0.17	0.04
Mg	12	K-series	0.06	0.08	0.08	0.04
		Total:	71.12	100.00	100.00	

Varnish+Plasma_3

PVP_1

El	AN	Series	unn. C [wt.%]	norm. C [wt.%]	Atom. C [at.%]	Error (1 Sigma) [wt.%]
Ca	20	K-series	28.03	35.81	25.23	0.88
O	8	K-series	26.94	34.42	38.30	4.92
P	15	K-series	11.33	14.48	10.06	0.48
C	6	K-series	8.07	12.50	17.89	2.18
N	7	K-series	1.70	0.00	0.00	0.00
Al	13	K-series	1.17	1.50	0.00	0.10
F	9	K-series	0.69	0.88	8.00	0.42
Na	11	K-series	0.16	0.21	0.49	0.05
Mg	12	K-series	0.13	0.16	0.01	0.04
Si	14	K-series	0.04	0.05	0.02	0.03
		Total:	78.27	100.00	100.00	

PVP_2

El	AN	Series	unn. C [wt.%]	norm. C [wt.%]	Atom. C [at.%]	Error (1 Sigma) [wt.%]
Ca	20	K-series	27.99	36.24	24.38	0.87
O	8	K-series	27.46	35.54	38.63	5.02
P	15	K-series	11.20	14.50	10.04	0.48
C	6	K-series	7.85	10.17	18.15	2.13
N	7	K-series	1.71	2.22	0.02	1.08
F	9	K-series	0.72	0.93	8.05	0.43
Na	11	K-series	0.16	0.21	0.50	0.05
Mg	12	K-series	0.13	0.16	0.09	0.04
Si	14	K-series	0.03	0.03	0.14	0.03
		Total:	77.24	100.00	100.00	

PVP_3

El	AN	Series	unn. C [wt.%]	norm. C [wt.%]	Atom. C [at.%]	Error (1 Sigma) [wt.%]
Ca	20	K-series	26.67	40.35	22.74	0.82
O	8	K-series	18.01	27.24	38.46	3.10
P	15	K-series	11.90	17.99	13.12	0.49
C	6	K-series	7.00	10.60	17.33	1.57
N	7	K-series	2.04	3.09	0.00	0.89
F	9	K-series	0.24	0.37	8.22	0.17
Na	11	K-series	0.17	0.27	0.08	0.04
Si	14	K-series	0.08	0.09	0.05	0.03
Mg	12	K-series	0.00	0.00	0.00	0.00
		Total:	66.11	100.00	100	
