# Supplementary material for: Combination of Cold Helium Plasma with Fluoride Varnish to Improve Enamel Surface Protection
Source: Materials (Basel). 2025 Sep 25;18(19):4466. doi: 10.3390/ma18194466 (PMC12525247; doi:10.3390/ma18194466)
Supplement: Supplementary file 1 [file materials-18-04466-s001.zip › Raw Result/EDX/EDX_Day1.pdf]

EDX_Day_1

Control_1

El	AN	Series	unn. C [wt.%]	norm. C [wt.%]	Atom. C [at.%]	Error (1 Sigma) [wt.%]
C	6	K-series	57.50	57.50	43.10	7.66
O	8	K-series	33.61	33.61	22.10	5.25
N	7	K-series	4.16	4.16	1.84	1.55
Si	14	K-series	1.73	1.73	0.08	0.11
P	15	K-series	1.30	1.30	12.57	0.09
Ca	20	K-series	0.83	0.83	20.28	0.07
F	9	K-series	0.73	0.73	0.02	0.36
Na	11	K-series	0.08	0.08	0.02	0.04
Mg	12	K-series	0.06	0.06	0.03	0.03
		Total:	100.00	100.00	100.00	

Control_2

El	AN	Series	unn. C [wt.%]	norm. C [wt.%]	Atom. C [at.%]	Error (1 Sigma) [wt.%]
C	6	K-series	57.16	57.16	42.82	8.40
O	8	K-series	35.24	35.24	20.00	6.20
N	7	K-series	3.10	3.10	1.02	1.67
Si	14	K-series	1.97	1.97	0.26	0.13
P	15	K-series	1.15	1.15	13.50	0.09
Ca	20	K-series	0.68	0.68	22.23	0.07
F	9	K-series	0.49	0.49	0.05	0.37
Na	11	K-series	0.14	0.14	0.08	0.05
Mg	12	K-series	0.06	0.06	0.04	0.04
		Total:	100.00	100.00	100.00	

Control_3

El	AN	Series	unn. C [wt.%]	norm. C [wt.%]	Atom. C [at.%]	Error (1 Sigma) [wt.%]
C	6	K-series	59.28	59.28	41.33	8.98
O	8	K-series	34.25	34.25	21.77	6.56
N	7	K-series	3.89	3.89	0.74	2.18
Si	14	K-series	1.29	1.29	0.61	0.10
P	15	K-series	0.71	0.71	13.31	0.08
Ca	20	K-series	0.38	0.38	22.13	0.07
Na	11	K-series	0.12	0.12	0.04	0.05
Mg	12	K-series	0.08	0.08	0.07	0.04
F	9	K-series	0.00	0.00	0.00	0.00
		Total:	100.00	100.00	100.00	

El	AN	Series	unn. C [wt.%]	norm. C [wt.%]	Atom. C [at.%]	Error (1 Sigma) [wt.%]
C	6	K-series	57.34	57.34	42.18	8.79
O	8	K-series	33.90	33.90	21.93	6.34
N	7	K-series	3.07	3.07	0.95	1.83
Si	14	K-series	2.08	2.08	0.03	0.14
F	9	K-series	1.31	1.31	0.04	0.70
P	15	K-series	1.30	1.30	13.57	0.10
Ca	20	K-series	0.83	0.83	21.28	0.08
Na	11	K-series	0.16	0.16	0.01	0.05
Mg	12	K-series	0.01	0.01	0.01	0.03
		Total:	100.00	100.00	100.00	

Helium gas_1

El	AN	Series	unn. C [wt.%]	norm. C [wt.%]	Atom. C [at.%]	Error (1 Sigma) [wt.%]
C	6	K-series	58.17	58.17	42.81	7.99
O	8	K-series	32.95	32.95	22.33	5.42
N	7	K-series	3.44	3.44	1.25	1.52
Si	14	K-series	1.74	1.74	0.84	0.11
F	9	K-series	1.41	1.41	0.01	0.57
P	15	K-series	1.04	1.04	12.46	0.08
Ca	20	K-series	0.71	0.71	20.24	0.07
Na	11	K-series	0.50	0.50	0.03	0.08
Mg	12	K-series	0.05	0.05	0.03	0.03
		Total:	100.00	100.00	100.00	

Helium gas_2

El	AN	Series	unn. C [wt.%]	norm. C [wt.%]	Atom. C [at.%]	Error (1 Sigma) [wt.%]
C	6	K-series	57.79	57.79	41.48	7.36
O	8	K-series	35.45	35.45	22.15	5.12
N	7	K-series	2.33	2.33	0.63	0.94
Si	14	K-series	1.81	1.81	0.88	0.11
P	15	K-series	1.10	1.10	13.48	0.08
Ca	20	K-series	0.89	0.89	21.30	0.07
F	9	K-series	0.56	0.56	0.04	0.27
Na	11	K-series	0.06	0.06	0.04	0.03
Mg	12	K-series	0.00	0.00	0.00	0.00
		Total:	100.00	100.00	100.00	

Helium gas_3

El	AN	Series	unn. C [wt.%]	norm. C [wt.%]	Atom. C [at.%]	Error (1 Sigma) [wt.%]
C	6	K-series	59.02	59.02	26.48	8.20
O	8	K-series	35.72	35.72	35.20	5.90
N	7	K-series	1.87	1.87	1.78	1.12
Si	14	K-series	1.72	1.72	0.83	0.11
P	15	K-series	0.85	0.85	13.37	0.07
Ca	20	K-series	0.71	0.71	22.24	0.07
Na	11	K-series	0.06	0.06	0.03	0.04
Mg	12	K-series	0.04	0.04	0.02	0.03
F	9	K-series	0.01	0.01	0.05	0.06
		Total:	100.00	100.00	100.00	

Plasma_1

El	AN	Series	unn. C [wt.%]	norm. C [wt.%]	Atom. C [at.%]	Error (1 Sigma) [wt.%]
C	6	K-series	59.61	59.61	22.78	8.09
O	8	K-series	35.31	35.31	36.07	5.73
N	7	K-series	2.30	2.30	5.95	1.21
Si	14	K-series	1.41	1.41	1.68	0.10
Ca	20	K-series	0.56	0.56	21.17	0.06
P	15	K-series	0.45	0.45	12.20	0.06
F	9	K-series	0.34	0.34	0.04	0.26
Na	11	K-series	0.05	0.05	0.06	0.03
Mg	12	K-series	0.05	0.05	0.05	0.03
		Total:	100.00	100.00	100.00	

Plasma_2

El	AN	Series	unn. C [wt.%]	norm. C [wt.%]	Atom. C [at.%]	Error (1 Sigma) [wt.%]
C	6	K-series	57.56	57.56	25.17	8.77
O	8	K-series	35.17	35.17	36.60	6.54
N	7	K-series	2.72	2.72	2.62	1.72
Si	14	K-series	1.72	1.72	1.83	0.12
P	15	K-series	0.89	0.89	12.39	0.08
F	9	K-series	0.81	0.81	0.08	0.54
Ca	20	K-series	0.68	0.68	21.23	0.08
Na	11	K-series	0.32	0.32	0.01	0.07
Mg	12	K-series	0.13	0.13	0.07	0.05
		Total:	100.00	100.00	100.00	

Plasma_3

El	AN	Series	unn. C [wt.%]	norm. C [wt.%]	Atom. C [at.%]	Error (1 Sigma) [wt.%]
C	6	K-series	58.86	58.86	35.13	7.61
O	8	K-series	35.80	35.80	27.85	5.25
N	7	K-series	3.04	3.04	0.08	1.20
Si	14	K-series	0.00	0.00	0.00	0.08
F	9	K-series	0.84	0.84	1.82	0.37
P	15	K-series	0.68	0.68	11.69	0.06
Ca	20	K-series	0.48	0.48	23.15	0.05
Na	11	K-series	0.25	0.25	0.25	0.05
Mg	12	K-series	0.05	0.05	0.03	0.03
		Total:	100.00	100.00	100.00	

Varnish_1

El	AN	Series	unn. C [wt.%]	norm. C [wt.%]	Atom. C [at.%]	Error (1 Sigma) [wt.%]
C	6	K-series	55.11	55.11	33.89	9.43
O	8	K-series	30.11	30.11	28.00	6.37
N	7	K-series	5.40	5.40	0.77	2.90
P	15	K-series	3.63	3.63	11.63	0.21
Si	14	K-series	3.12	3.12	1.05	0.19
Ca	20	K-series	1.46	1.46	22.51	0.12
F	9	K-series	1.24	1.24	1.89	0.73
Mg	12	K-series	0.03	0.03	0.02	0.03
Na	11	K-series	0.10	0.10	0.24	0.03
		Total:	100.00	100.00	100.00	

Varnish_2

El	AN	Series	unn. C [wt.%]	norm. C [wt.%]	Atom. C [at.%]	Error (1 Sigma) [wt.%]
C	6	K-series	56.10	56.10	33.79	8.46
O	8	K-series	35.48	35.48	27.29	6.41
N	7	K-series	3.71	3.71	1.01	1.94
Si	14	K-series	2.00	2.00	0.97	0.13
P	15	K-series	1.33	1.33	11.59	0.10
F	9	K-series	0.62	0.62	1.85	0.48
Ca	20	K-series	0.65	0.65	23.22	0.07
Mg	12	K-series	0.02	0.02	0.01	0.03
Na	11	K-series	0.10	0.10	0.27	0.10
		Total:	100.00	100.00	100.00	

Varnish_3

El	AN	Series	unn. C [wt.%]	norm. C [wt.%]	Atom. C [at.%]	Error (1 Sigma) [wt.%]
C	6	K-series	56.92	56.92	17.79	8.73
O	8	K-series	31.87	31.87	38.05	6.60
N	7	K-series	2.60	2.60	1.74	1.66
Si	14	K-series	2.01	2.01	0.98	0.14
P	15	K-series	1.27	1.27	11.56	0.10
Ca	20	K-series	1.21	1.21	24.41	0.10
Na	11	K-series	0.07	0.07	0.19	0.04
Mg	12	K-series	0.04	0.04	0.06	0.03
F	9	K-series	4.00	4.00	5.22	2.00
		Total:	100.00	100.00	100.00	

Plasma+Varnish_1

El	AN	Series	unn. C [wt.%]	norm. C [wt.%]	Atom. C [at.%]	Error (1 Sigma) [wt.%]
C	6	K-series	57.04	57.04	19.50	8.43
O	8	K-series	34.95	34.95	37.67	6.31
N	7	K-series	3.74	3.74	0.56	1.94
P	14	K-series	1.72	1.72	11.83	0.12
F	9	K-series	1.15	1.15	4.90	0.63
Ca	20	K-series	1.13	1.13	25.38	0.10
Na	11	K-series	0.20	0.20	0.12	0.06
Mg	12	K-series	0.07	0.07	0.04	0.04
		Total:	100.00	100.00	100.00	

Plasma+Varnish_2

El	AN	Series	unn. C [wt.%]	norm. C [wt.%]	Atom. C [at.%]	Error (1 Sigma) [wt.%]
C	6	K-series	59.51	59.51	18.76	7.80
O	8	K-series	34.74	34.74	37.62	5.37
N	7	K-series	2.73	2.73	1.83	1.19
Si	14	K-series	1.28	1.28	0.62	0.09
Ca	20	K-series	0.75	0.75	24.25	0.07
P	15	K-series	0.74	0.74	11.20	0.07
F	9	K-series	0.17	0.17	5.60	0.17
Na	11	K-series	0.04	0.04	0.10	0.03
Mg	12	K-series	0.03	0.03	0.02	0.03
		Total:	100.00	100.00	100.0	

Plasma+Varnish_3

El	AN	Series	unn. C [wt.%]	norm. C [wt.%]	Atom. C [at.%]	Error (1 Sigma) [wt.%]
Ca	20	K-series	31.15	44.55	24.05	0.97
P	15	K-series	16.38	26.29	12.54	0.77
O	8	K-series	12.81	16.32	36.36	3.42
C	6	K-series	5.09	7.28	19.39	1.90
N	7	K-series	1.95	2.79	3.43	1.48
Si	14	K-series	0.24	0.34	0.31	0.05
Na	11	K-series	0.21	0.31	0.34	0.06
Mg	12	K-series	0.08	0.11	0.11	0.04
F	9	K-series	2.00	2.00	3.47	1.05
		Total:	69.92	100.00	100.00	

Varnish+Plasma_1

El	AN	Series	unn. C [wt.%]	norm. C [wt.%]	Atom. C [at.%]	Error (1 Sigma) [wt.%]
Ca	20	K-series	24.96	46.53	23.08	0.79
P	15	K-series	12.06	22.47	11.42	0.52
C	6	K-series	6.77	12.63	18.52	2.11
O	8	K-series	6.37	11.87	37.02	2.01
N	7	K-series	3.01	5.61	3.73	1.73
Si	14	K-series	0.16	0.30	1.74	0.04
F	9	K-series	0.15	0.28	3.97	0.20
Na	11	K-series	0.09	0.16	0.37	0.04
Mg	12	K-series	0.08	0.15	0.15	0.04
		Total:	53.65	100.00	100.00	

Varnish+Plasma_2

El	AN	Series	unn. C [wt.%]	norm. C [wt.%]	Atom. C [at.%]	Error (1 Sigma) [wt.%]
Ca	20	K-series	27.34	40.03	24.53	0.84
O	8	K-series	17.65	26.77	37.21	3.43
P	15	K-series	12.92	18.91	12.33	0.54
C	6	K-series	5.87	8.59	17.39	1.47
N	7	K-series	1.93	2.85	3.96	0.93
Na	11	K-series	0.39	0.57	0.39	0.06
Si	14	K-series	0.11	0.16	0.13	0.03
Mg	12	K-series	0.06	0.08	0.08	0.03
F	9	K-series	2.04	2.04	3.98	1.51
		Total:	68.30	100.00	100.00	

Varnish+Plasma_3

El	AN	Series	unn. C [wt.%]	norm. C [wt.%]	Atom. C [at.%]	Error (1 Sigma) [wt.%]
O	8	K-series	34.32	42.77	38.02	5.17
Ca	20	K-series	25.57	31.87	25.37	0.79
P	15	K-series	10.34	12.89	10.50	0.43
C	6	K-series	7.35	9.17	17.50	1.68
N	7	K-series	1.86	2.31	0.56	0.84
Na	11	K-series	0.34	0.42	0.47	0.06
F	9	K-series	0.32	0.40	7.54	0.22
Mg	12	K-series	0.10	0.13	0.03	0.04
Si	14	K-series	0.03	0.04	0.01	0.03
		Total:	80.23	100.00	100.00	

PVP_1

El	AN	Series	unn. C [wt.%]	norm. C [wt.%]	Atom. C [at.%]	Error (1 Sigma) [wt.%]
Ca	20	K-series	25.24	39.63	24.02	0.80
O	8	K-series	21.21	33.35	38.43	4.31
P	15	K-series	8.60	14.16	10.07	0.42
C	6	K-series	6.60	10.36	15.21	2.05
Na	11	K-series	0.33	0.52	0.50	0.07
Mg	12	K-series	0.24	0.37	1.34	0.05
F	9	K-series	0.24	0.37	7.59	0.25
Si	14	K-series	1.03	1.03	2.50	0.97
N	7	K-series	0.20	0.20	0.40	0.03
		Total:	63.67	100.00	100.00	

PVP_2

El	AN	Series	unn. C [wt.%]	norm. C [wt.%]	Atom. C [at.%]	Error (1 Sigma) [wt.%]
Ca	20	K-series	27.48	43.31	24.57	0.86
O	8	K-series	17.26	27.20	37.60	3.69
P	15	K-series	11.13	17.55	10.20	0.47
C	6	K-series	5.95	9.37	17.17	1.85
N	7	K-series	1.14	1.79	0.32	0.92
F	9	K-series	0.33	0.52	7.94	0.28
Na	11	K-series	0.08	0.12	0.08	0.04
Mg	12	K-series	0.07	0.11	1.10	0.04
Si	14	K-series	0.02	0.03	1.02	0.03
		Total:	63.45	100.00	100.00	

PVP_3
