# Supplementary material for: Combination of Cold Helium Plasma with Fluoride Varnish to Improve Enamel Surface Protection
Source: Materials (Basel). 2025 Sep 25;18(19):4466. doi: 10.3390/ma18194466 (PMC12525247; doi:10.3390/ma18194466)
Supplement: Supplementary file 1 [file materials-18-04466-s001.zip › Raw Result/EDX/EDX_Day2.pdf]

EDX_Day_2

Control_1

El	AN	Series	unn. C [wt.%]	norm. C [wt.%]	Atom. C [at.%]	Error (1 Sigma) [wt.%]
C	6	K-series	55.19	55.19	43.09	8.78
O	8	K-series	34.26	34.26	22.04	6.57
N	7	K-series	5.09	5.09	1.44	2.48
Si	14	K-series	2.53	2.53	0.34	0.16
P	15	K-series	1.51	1.51	12.67	0.11
Ca	20	K-series	1.00	1.00	20.34	0.08
F	9	K-series	0.21	0.21	0.01	0.28
Na	11	K-series	0.11	0.11	0.02	0.05
Mg	12	K-series	0.09	0.09	0.05	0.04
		Total:	100.00	100.00	100.00	

Control_2

El	AN	Series	unn. C [wt.%]	norm. C [wt.%]	Atom. C [at.%]	Error (1 Sigma) [wt.%]
C	6	K-series	57.81	57.81	42.36	7.20
O	8	K-series	31.19	31.19	20.17	4.47
N	7	K-series	6.03	6.03	1.39	1.57
Si	14	K-series	2.11	2.11	0.02	0.12
P	15	K-series	1.31	1.31	13.58	0.08
Ca	20	K-series	0.79	0.79	22.27	0.06
F	9	K-series	0.41	0.41	0.04	0.20
S	16	K-series	0.17	0.17	0.07	0.03
Mg	12	K-series	0.12	0.12	0.06	0.04
Na	11	K-series	0.06	0.06	0.04	0.03
		Total:	100.00	100.00	100.00	

Control_3

El	AN	Series	unn. C [wt.%]	norm. C [wt.%]	Atom. C [at.%]	Error (1 Sigma) [wt.%]
C	6	K-series	59.51	59.51	41.01	7.97
O	8	K-series	34.88	34.88	21.40	5.53
N	7	K-series	2.37	2.37	1.23	1.16
Si	14	K-series	1.64	1.64	0.79	0.11
P	15	K-series	0.79	0.79	13.35	0.07
Ca	20	K-series	0.47	0.47	22.16	0.06
F	9	K-series	0.23	0.23	0.01	0.21
Na	11	K-series	0.09	0.09	0.05	0.04
Mg	12	K-series	0.00	0.00	0.00	0.00
		Total:	100.00	100.00	100.00	

El	AN	Series	unn. C [wt.%]	norm. C [wt.%]	Atom. C [at.%]	Error (1 Sigma) [wt.%]
C	6	K-series	59.51	59.51	42.16	7.80
O	8	K-series	34.74	34.74	21.26	5.37
N	7	K-series	2.73	2.73	1.13	1.19
Si	14	K-series	1.28	1.28	0.62	0.09
Ca	20	K-series	0.75	0.75	21.25	0.07
P	15	K-series	0.74	0.74	13.52	0.07
F	9	K-series	0.17	0.17	0.02	0.17
Na	11	K-series	0.04	0.04	0.02	0.03
Mg	12	K-series	0.03	0.03	0.02	0.03
		Total:	100.00	100.00	100	

Helium gas_1

El	AN	Series	unn. C [wt.%]	norm. C [wt.%]	Atom. C [at.%]	Error (1 Sigma) [wt.%]
C	6	K-series	55.78	55.78	42.79	9.50
O	8	K-series	29.98	29.98	22.14	6.58
N	7	K-series	7.46	7.46	1.10	3.58
Si	14	K-series	2.76	2.76	0.53	0.17
P	15	K-series	1.94	1.94	12.86	0.13
Ca	20	K-series	1.42	1.42	20.49	0.11
F	9	K-series	0.55	0.55	0.01	0.52
Mg	12	K-series	0.09	0.09	0.05	0.04
Na	11	K-series	0.01	0.01	0.03	0.03
		Total:	100.00	100.00	100.00	

Helium gas_2

El	AN	Series	unn. C [wt.%]	norm. C [wt.%]	Atom. C [at.%]	Error (1 Sigma) [wt.%]
C	6	K-series	58.10	58.10	41.41	8.75
O	8	K-series	31.34	31.34	22.07	5.85
N	7	K-series	4.87	4.87	1.25	2.18
Si	14	K-series	2.56	2.56	0.24	0.15
P	15	K-series	1.15	1.15	13.55	0.08
Ca	20	K-series	1.12	1.12	21.35	0.08
F	9	K-series	0.77	0.77	0.05	0.49
Na	11	K-series	0.09	0.09	0.05	0.04
Mg	12	K-series	0.01	0.01	0.03	0.03
		Total:	100.00	100.00	100.00	

Helium gas_3

El	AN	Series	unn. C [wt.%]	norm. C [wt.%]	Atom. C [at.%]	Error (1 Sigma) [wt.%]
C	6	K-series	56.47	56.47	25.92	10.02
O	8	K-series	26.61	26.61	35.00	6.44
N	7	K-series	8.34	8.34	2.38	4.14
Ca	20	K-series	2.84	2.84	21.98	0.16
P	15	K-series	2.79	2.79	13.25	0.17
Si	14	K-series	2.67	2.67	1.30	0.17
F	9	K-series	0.24	0.24	0.07	0.36
Na	11	K-series	0.08	0.08	0.05	0.05
Mg	12	K-series	0.04	0.04	0.05	0.03
		Total:	100.00	100.00	100.00	

Plasma_1

El	AN	Series	unn. C [wt.%]	norm. C [wt.%]	Atom. C [at.%]	Error (1 Sigma) [wt.%]
C	6	K-series	57.97	57.97	22.41	7.87
O	8	K-series	32.95	32.95	27.92	5.34
N	7	K-series	4.71	4.71	9.03	1.74
Si	14	K-series	1.61	1.61	6.78	0.10
Ca	20	K-series	1.01	1.01	21.34	0.07
P	15	K-series	0.92	0.92	12.40	0.07
F	9	K-series	0.81	0.81	0.04	0.40
Na	11	K-series	0.01	0.01	0.05	0.03
Mg	12	K-series	0.01	0.01	0.03	0.03
		Total:	100.00	100.00	100.00	

Plasma_2

El	AN	Series	unn. C [wt.%]	norm. C [wt.%]	Atom. C [at.%]	Error (1 Sigma) [wt.%]
C	6	K-series	54.79	54.79	24.93	8.98
O	8	K-series	32.08	32.08	36.55	6.49
N	7	K-series	5.57	5.57	3.07	2.77
Si	14	K-series	2.35	2.35	1.15	0.15
F	9	K-series	1.90	1.90	0.08	0.95
P	15	K-series	1.48	1.48	12.66	0.11
Ca	20	K-series	1.39	1.39	21.48	0.10
Na	11	K-series	0.25	0.25	0.05	0.07
Mg	12	K-series	0.18	0.18	0.03	0.05
		Total:	100.00	100.00	100.00	

Plasma_3

El	AN	Series	unn. C [wt.%]	norm. C [wt.%]	Atom. C [at.%]	Error (1 Sigma) [wt.%]
C	6	K-series	59.35	59.35	35.51	8.00
O	8	K-series	34.77	34.77	27.05	5.59
N	7	K-series	3.07	3.07	0.41	1.36
Si	14	K-series	1.45	1.45	0.70	0.10
Ca	20	K-series	0.50	0.50	23.10	0.06
P	15	K-series	0.52	0.52	11.72	0.06
F	9	K-series	0.20	0.20	1.19	0.20
Na	11	K-series	0.04	0.04	0.22	0.03
Mg	12	K-series	0.09	0.09	0.10	0.04
		Total:	100.00	100.00	100.00	

Varnish_1

El	AN	Series	unn. C [wt.%]	norm. C [wt.%]	Atom. C [at.%]	Error (1 Sigma) [wt.%]
C	6	K-series	57.58	57.58	33.58	8.50
O	8	K-series	30.35	30.35	27.95	5.57
N	7	K-series	5.58	5.58	1.38	2.32
Si	14	K-series	2.28	2.28	1.11	0.14
Ca	20	K-series	1.78	1.78	22.58	0.10
P	15	K-series	1.51	1.51	11.70	0.10
F	9	K-series	0.76	0.76	1.24	0.46
Mg	12	K-series	0.12	0.12	0.27	0.04
Na	11	K-series	0.05	0.05	0.19	0.04
		Total:	100.00	100.00	100.00	

Varnish_2

El	AN	Series	unn. C [wt.%]	norm. C [wt.%]	Atom. C [at.%]	Error (1 Sigma) [wt.%]
C	6	K-series	59.51	59.51	32.93	7.60
O	8	K-series	33.94	33.94	27.06	5.05
N	7	K-series	2.93	2.93	3.00	1.11
Ca	20	K-series	1.35	1.35	23.05	0.08
Si	14	K-series	1.08	1.08	0.52	0.08
P	15	K-series	0.79	0.79	11.64	0.06
F	9	K-series	0.35	0.35	1.21	0.22
Na	11	K-series	0.04	0.04	0.23	0.03
Mg	12	K-series	0.01	0.01	0.36	0.03
		Total:	100.00	100.00	100.00	

Varnish_3

El	AN	Series	unn. C [wt.%]	norm. C [wt.%]	Atom. C [at.%]	Error (1 Sigma) [wt.%]
C	6	K-series	56.83	56.83	17.22	8.62
O	8	K-series	34.10	34.10	37.93	6.34
N	7	K-series	5.15	5.15	3.19	2.32
Si	14	K-series	1.54	1.54	0.76	0.11
Ca	20	K-series	1.16	1.16	24.39	0.08
F	9	K-series	0.62	0.62	4.77	0.45
P	15	K-series	0.48	0.48	11.51	0.06
Na	11	K-series	0.08	0.08	0.15	0.04
Mg	12	K-series	0.04	0.04	0.08	0.03
		Total:	100.00	100.00	100.00	

Plasma+Varnish_1

El	AN	Series	unn. C [wt.%]	norm. C [wt.%]	Atom. C [at.%]	Error (1 Sigma) [wt.%]
O	8	K-series	25.65	36.07	37.05	4.54
Ca	20	K-series	22.17	31.18	25.70	0.70
P	15	K-series	9.84	13.84	12.02	0.42
C	6	K-series	9.03	12.70	19.34	2.19
N	7	K-series	3.74	5.27	1.08	1.55
Na	11	K-series	0.34	0.48	0.12	0.06
F	9	K-series	0.15	0.21	4.48	0.18
Mg	12	K-series	0.11	0.15	0.13	0.04
Si	14	K-series	0.08	0.11	0.08	0.03
		Total:	71.11	100.00	100.00	

Plasma+Varnish_2

El	AN	Series	unn. C [wt.%]	norm. C [wt.%]	Atom. C [at.%]	Error (1 Sigma) [wt.%]
O	8	K-series	35.89	41.48	37.12	6.03
Ca	20	K-series	25.36	29.32	24.32	0.80
P	15	K-series	11.08	12.81	11.27	0.47
C	6	K-series	10.70	12.37	18.58	2.60
N	7	K-series	1.50	1.73	0.16	0.96
F	9	K-series	1.24	1.43	5.12	0.59
Na	11	K-series	0.48	0.55	0.18	0.08
Mg	12	K-series	0.21	0.24	2.20	0.05
Si	14	K-series	0.06	0.07	1.05	0.03
		Total:	86.52	100.00	100.00	

Plasma+Varnish_3

Varnish+Plasma_1

El	AN	Series	unn. C [wt.%]	norm. C [wt.%]	Atom. C [at.%]	Error (1 Sigma) [wt.%]
O	8	K-series	29.44	36.06	35.83	4.98
Ca	20	K-series	23.43	28.92	24.20	0.77
C	6	K-series	11.62	14.23	19.56	2.60
P	15	K-series	10.47	12.83	12.54	0.44
N	7	K-series	3.17	3.89	0.21	1.38
F	9	K-series	1.61	1.98	3.03	0.63
Na	11	K-series	0.56	0.69	0.30	0.08
Mg	12	K-series	0.26	0.32	2.26	0.05
Si	14	K-series	1.08	1.09	2.07	1.03
		Total:	81.65	100.00	100.00	

Varnish+Plasma_2

El	AN	Series	unn. C [wt.%]	norm. C [wt.%]	Atom. C [at.%]	Error (1 Sigma) [wt.%]
C	6	K-series	50.99	50.99	17.72	8.09
O	8	K-series	34.70	34.70	36.52	5.72
N	7	K-series	2.89	2.89	5.81	1.38
Si	14	K-series	1.67	1.67	1.49	0.11
Ca	20	K-series	14.39	14.39	23.07	0.09
P	15	K-series	1.01	1.01	11.45	0.08
F	9	K-series	2.23	2.23	3.47	0.22
Mg	12	K-series	0.08	0.08	0.15	0.04
Na	11	K-series	0.04	0.04	0.32	0.03
		Total:	100.00	100.00	100.00	

Varnish+Plasma_3

El	AN	Series	unn. C [wt.%]	norm. C [wt.%]	Atom. C [at.%]	Error (1 Sigma) [wt.%]
O	8	K-series	32.17	41.38	37.07	5.88
Ca	20	K-series	20.85	26.82	24.09	0.68
C	6	K-series	10.13	13.02	17.20	2.72
P	15	K-series	9.95	12.80	12.08	0.44
N	7	K-series	2.35	3.02	5.51	1.39
F	9	K-series	1.51	1.95	3.49	0.74
Na	11	K-series	0.56	0.71	0.31	0.09
Mg	12	K-series	0.21	0.27	0.22	0.05
Si	14	K-series	0.03	0.04	0.03	0.03
		Total:	77.76	100.00	100.00	

El	AN	Series	unn. C [wt.%]	norm. C [wt.%]	Atom. C [at.%]	Error (1 Sigma) [wt.%]
O	8	K-series	36.14	44.06	38.14	6.23
Ca	20	K-series	20.11	23.78	25.48	0.79
P	15	K-series	10.46	12.44	10.54	0.45
C	6	K-series	8.91	10.54	16.62	2.31
N	7	K-series	2.40	2.85	0.35	1.26
Na	11	K-series	0.15	0.18	0.46	0.05
Mg	12	K-series	0.10	0.11	0.19	0.04
Si	14	K-series	0.00	0.00	1.05	0.00
F	9	K-series	6.03	6.03	7.17	0.00
		Total:	84.30	100.00	100.00	

PVP_1

El	AN	Series	unn. C [wt.%]	norm. C [wt.%]	Atom. C [at.%]	Error (1 Sigma) [wt.%]
O	8	K-series	31.09	37.00	38.14	5.72
Ca	20	K-series	22.01	32.02	24.18	4.82
P	15	K-series	10.73	13.62	10.17	0.46
C	6	K-series	7.46	9.53	14.88	2.11
N	7	K-series	0.68	0.86	0.67	0.64
Na	11	K-series	0.34	0.44	0.40	0.06
F	9	K-series	5.21	5.26	7.22	0.23
Mg	12	K-series	0.06	0.07	2.26	0.03
Si	14	K-series	1.20	1.20	2.08	0.90
		Total:	78.77	100.00	100.00	

PVP_2

El	AN	Series	unn. C [wt.%]	norm. C [wt.%]	Atom. C [at.%]	Error (1 Sigma) [wt.%]
Ca	20	K-series	28.03	35.81	24.53	0.88
O	8	K-series	26.94	34.42	37.30	4.92
P	15	K-series	11.33	14.48	10.26	0.48
C	6	K-series	8.07	10.32	17.09	2.18
N	7	K-series	1.70	2.18	0.69	1.07
Al	13	K-series	1.17	1.50	1.20	0.10
F	9	K-series	0.69	0.88	7.55	0.42
Na	11	K-series	0.16	0.21	0.20	0.05
Mg	12	K-series	0.13	0.16	0.14	0.04
Si	14	K-series	0.04	0.05	1.04	0.03
		Total:	78.27	100.00	100.00	

PVP_3
