# Supplementary figures and images for: Combination of Cold Helium Plasma with Fluoride Varnish to Improve Enamel Surface Protection
Source: Materials (Basel). 2025 Sep 25;18(19):4466. doi: 10.3390/ma18194466 (PMC12525247; doi:10.3390/ma18194466)

1

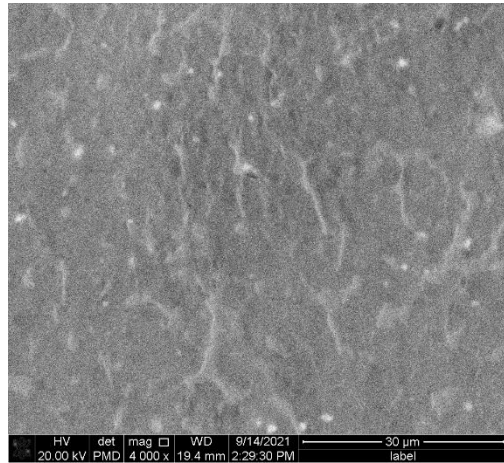

2

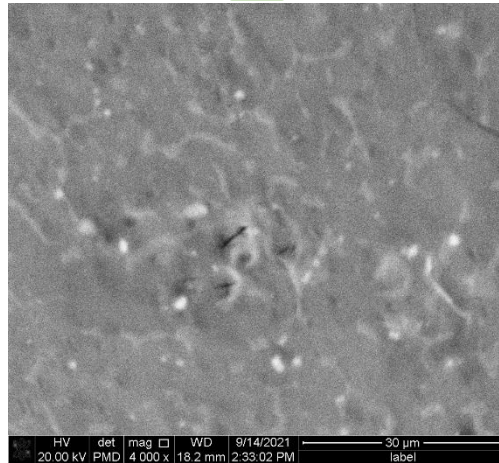

3

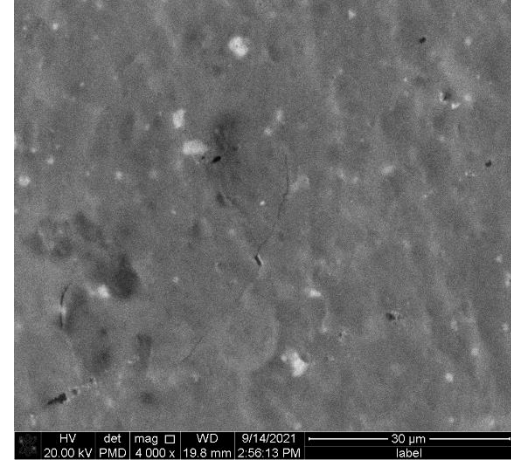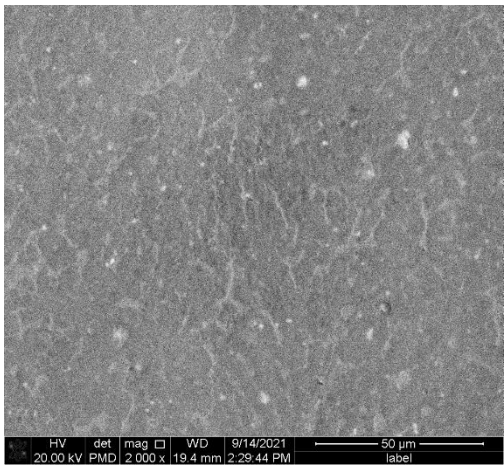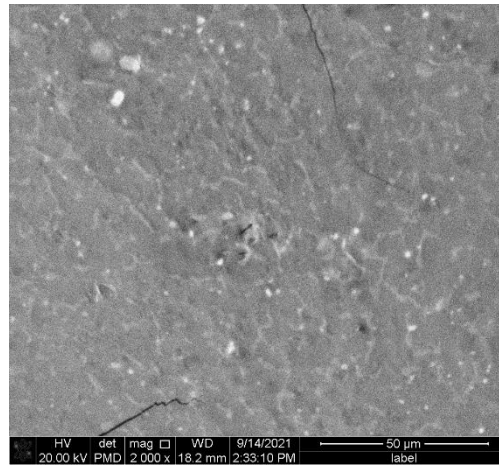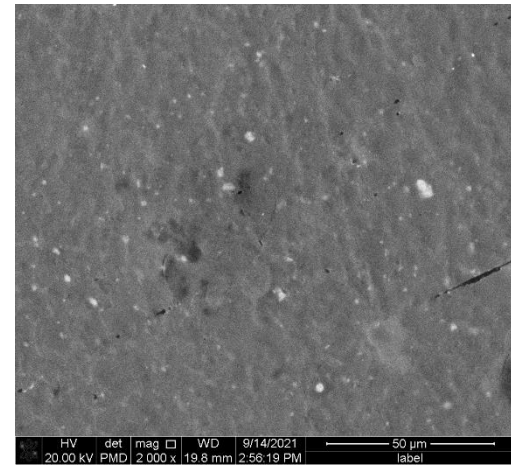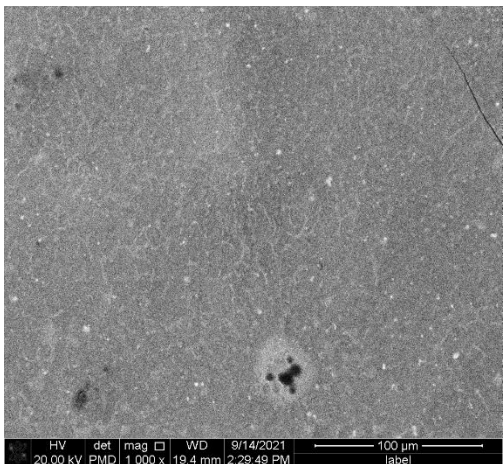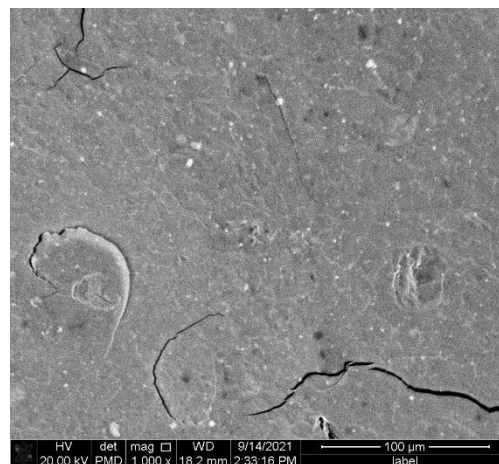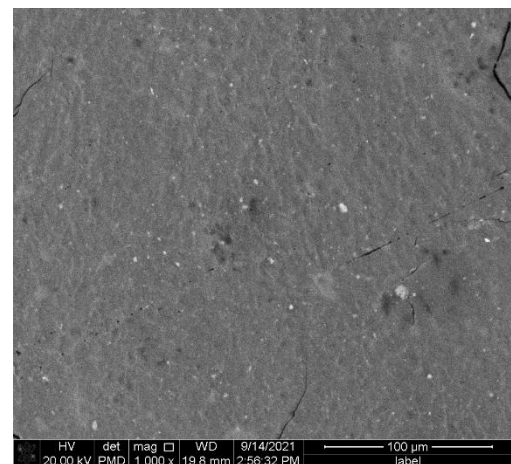

Plasma + Varnish

1

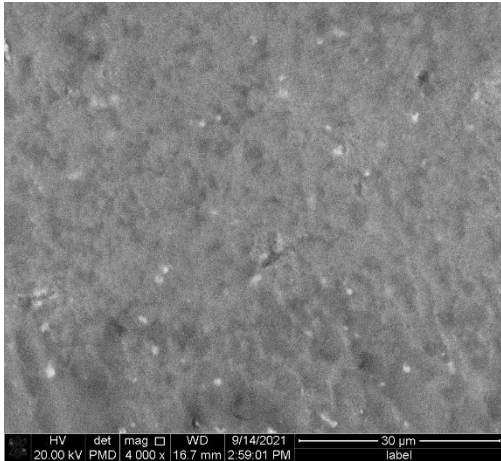

2

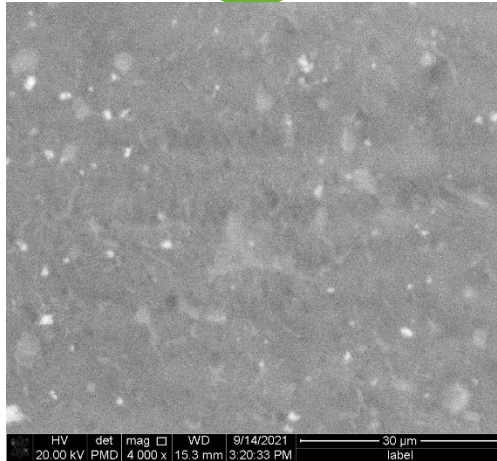

3

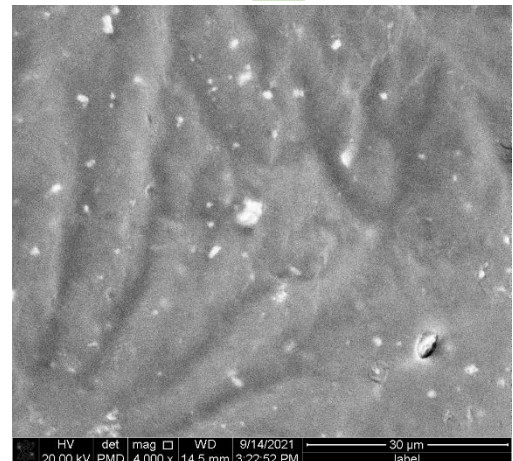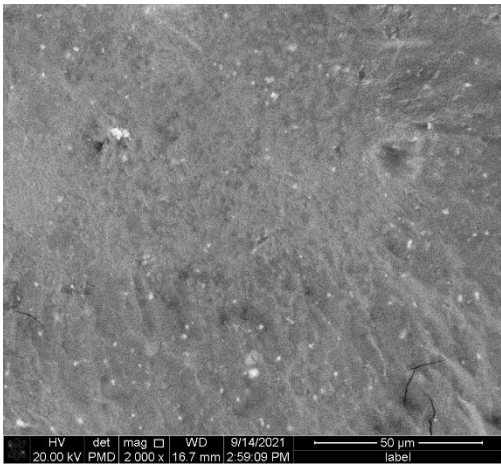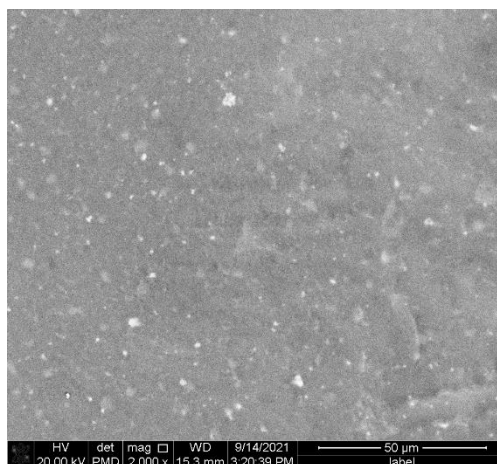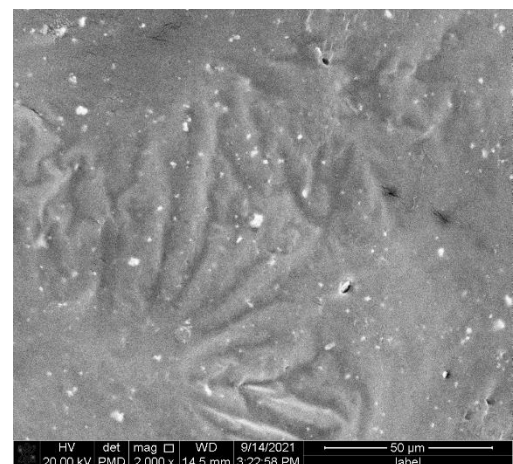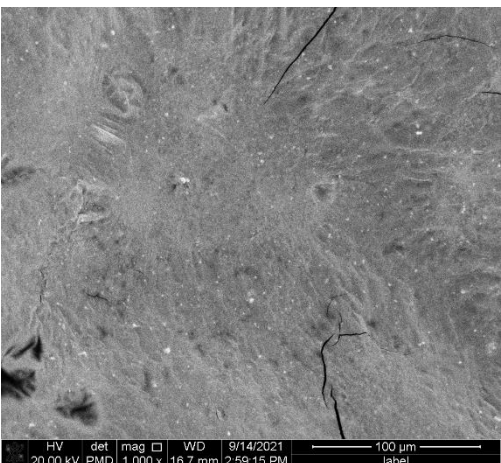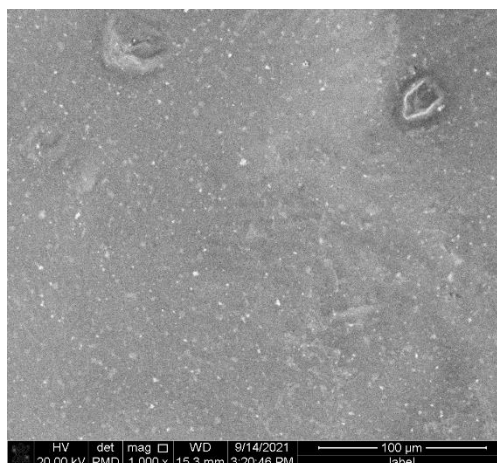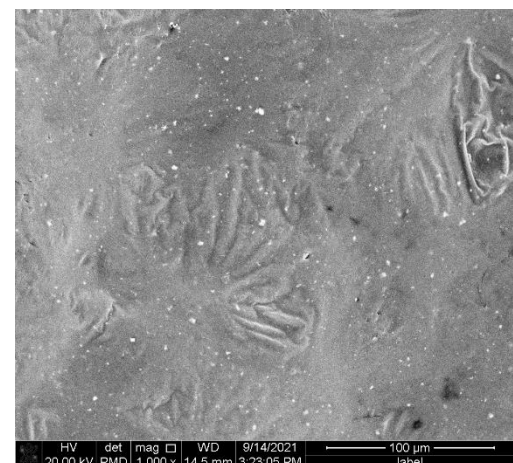

Plasma + Varnish + Plasma

1

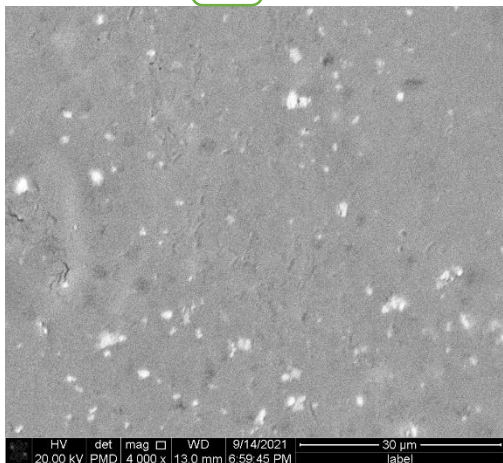

2

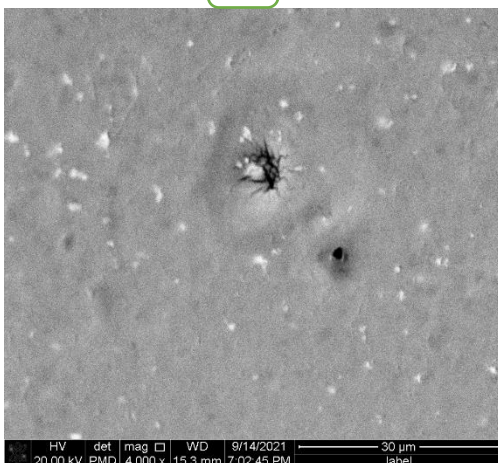

3

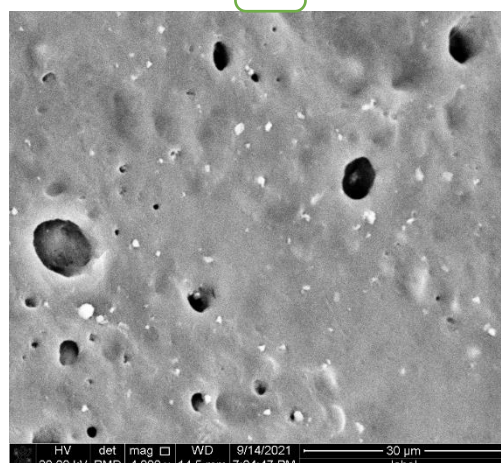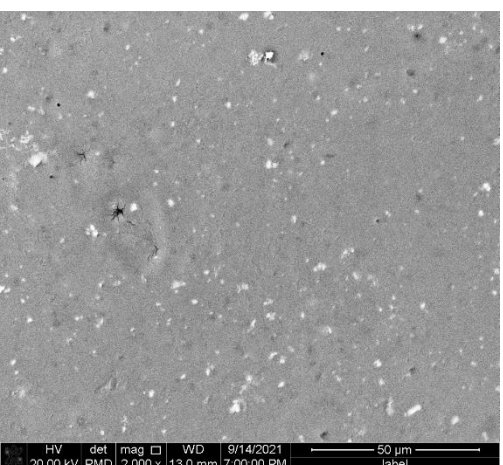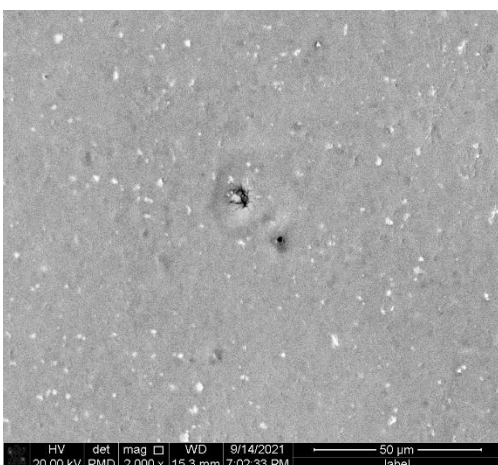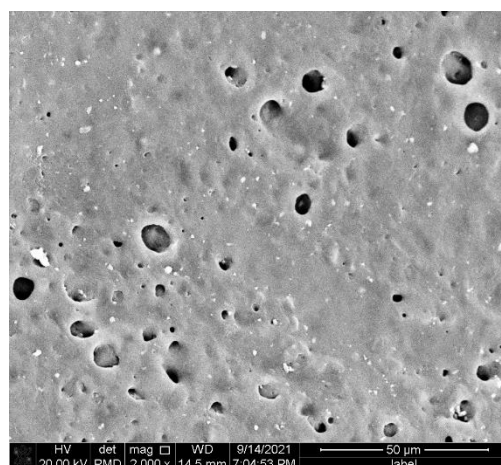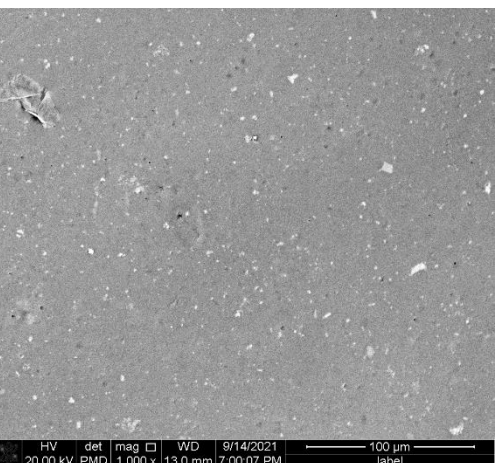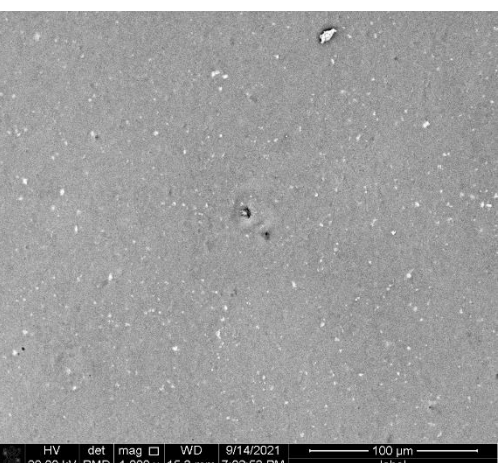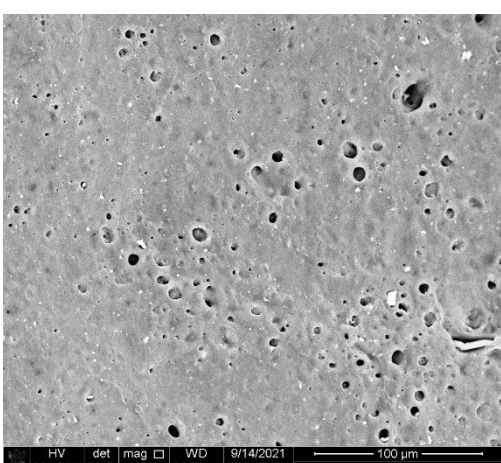

Varnish + Plasma

1

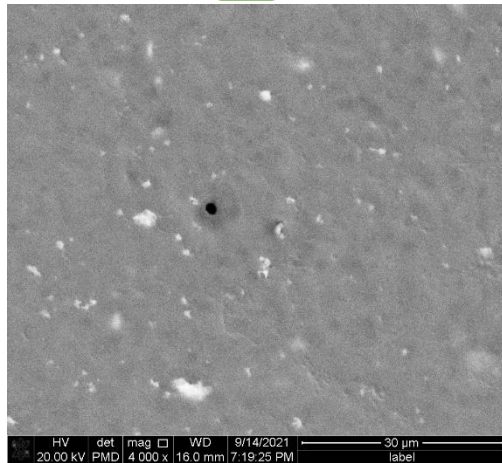

2

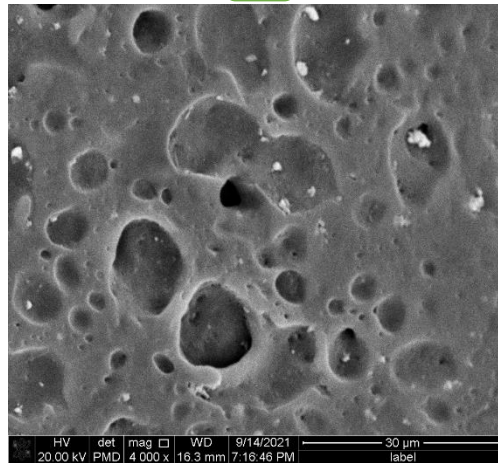

3

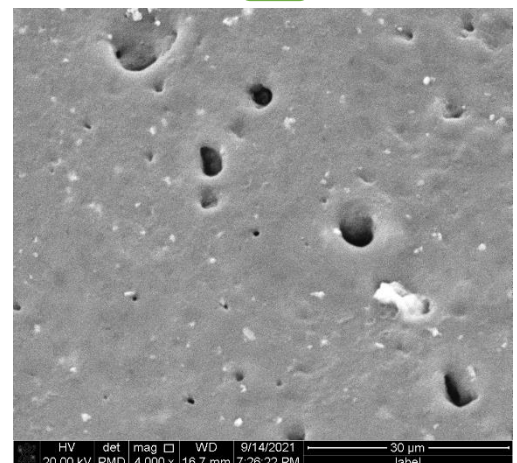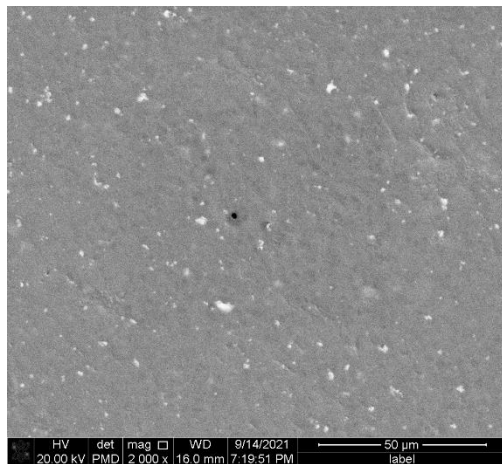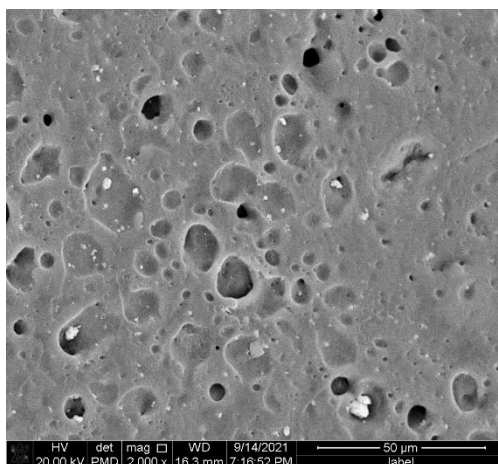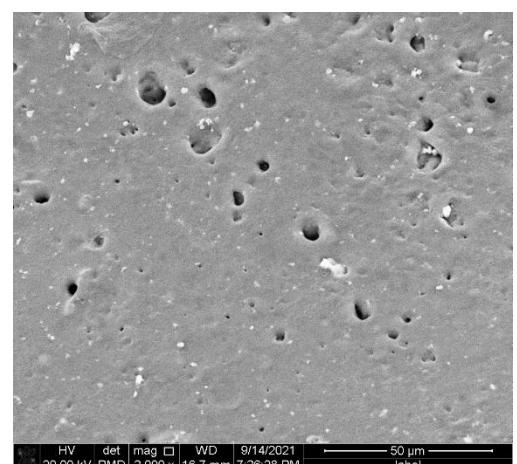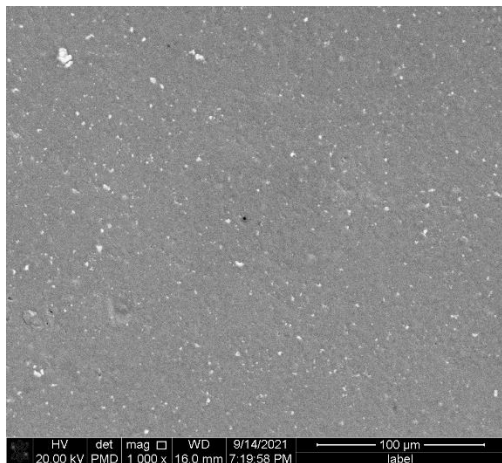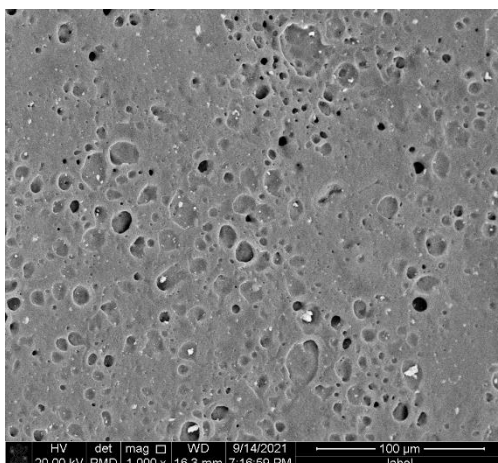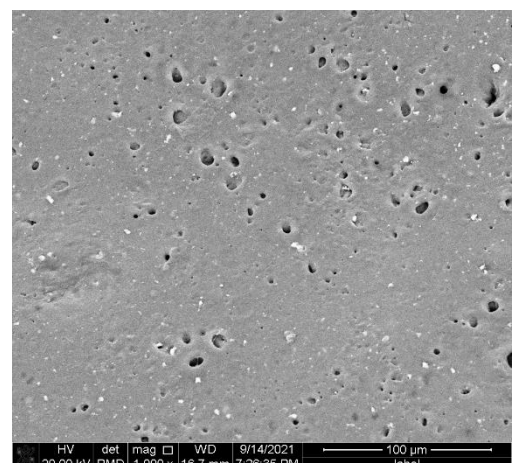

Varnish

1

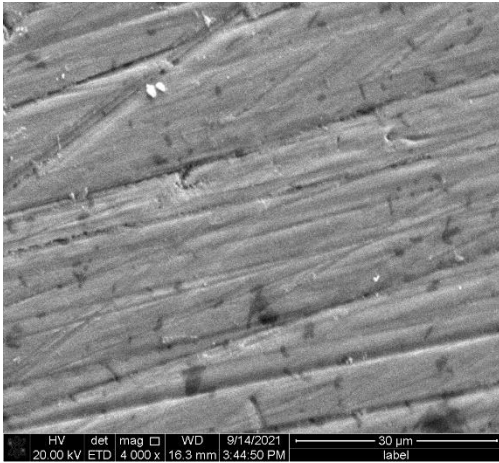

2

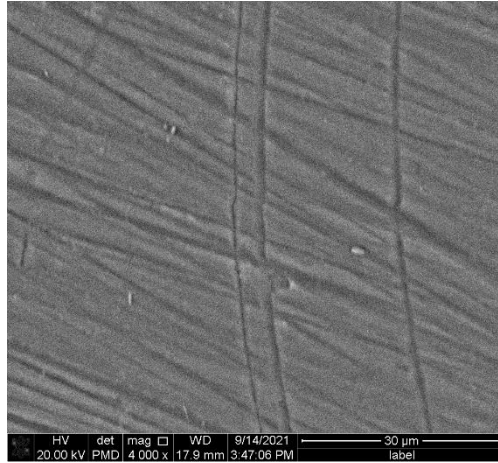

3

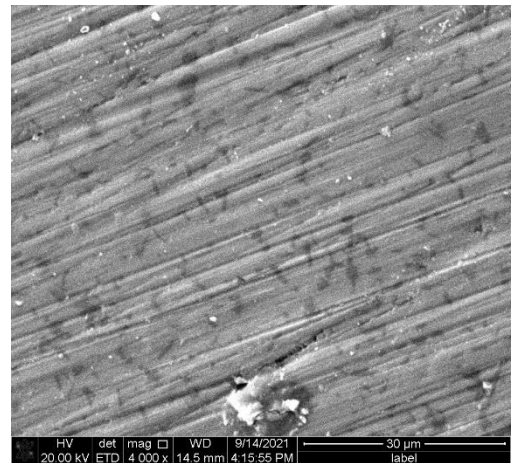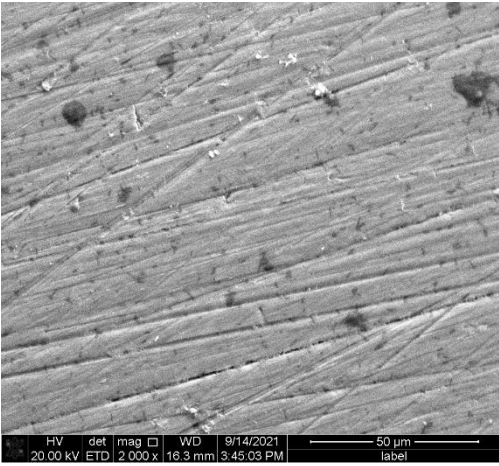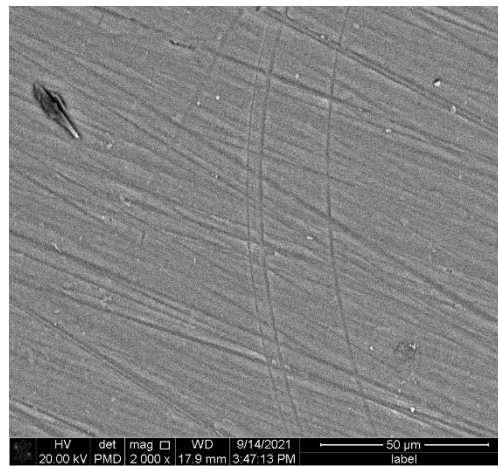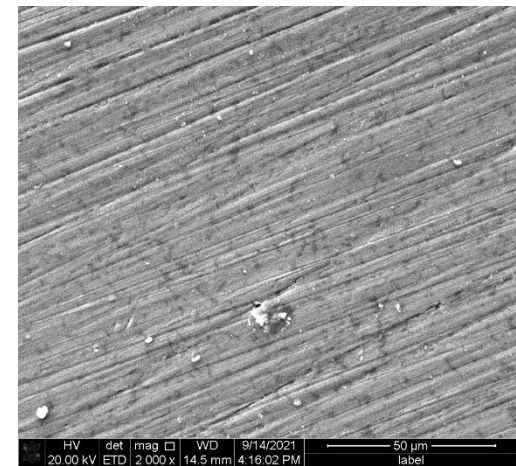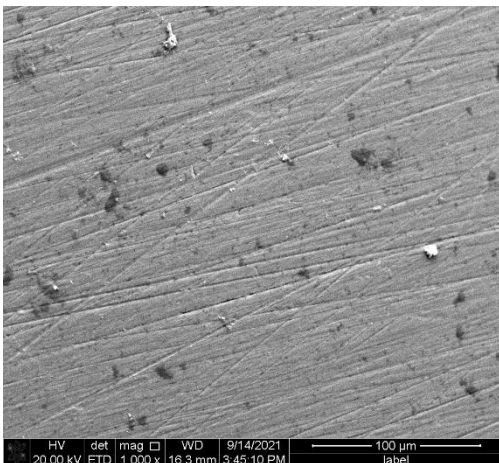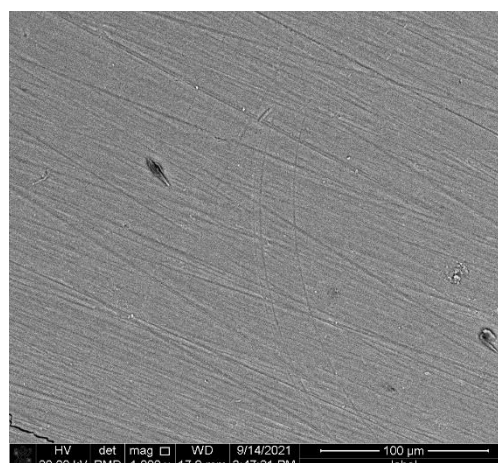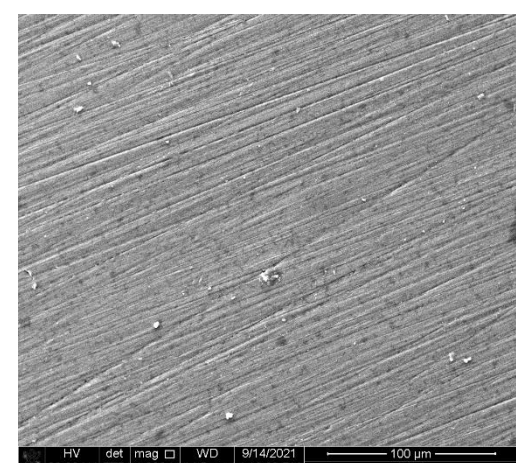

Plasma

1

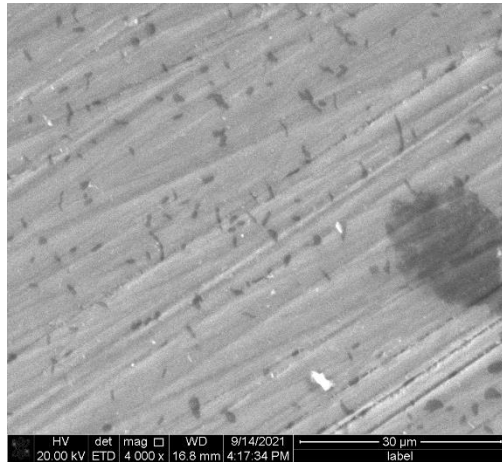

2

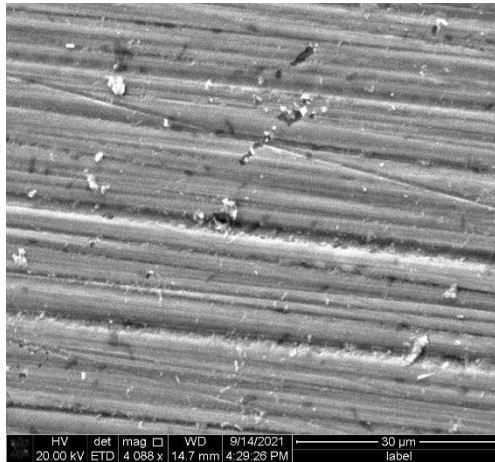

3

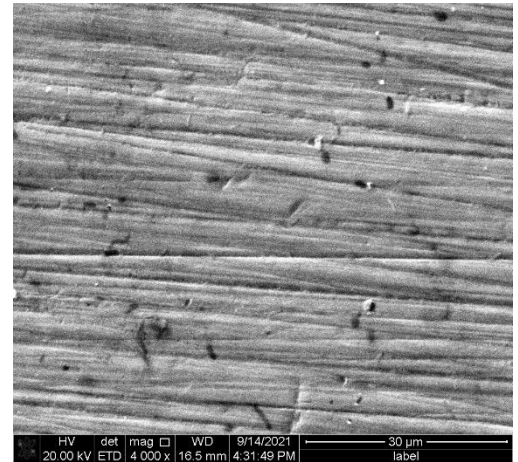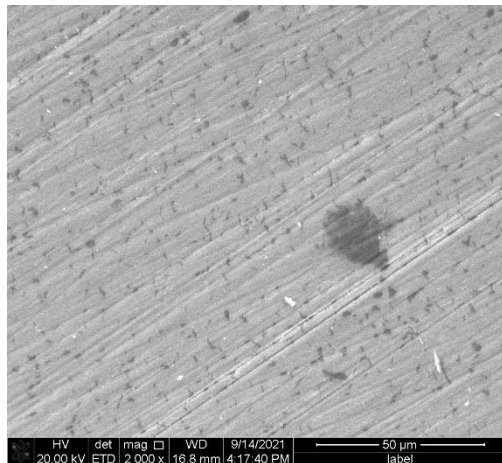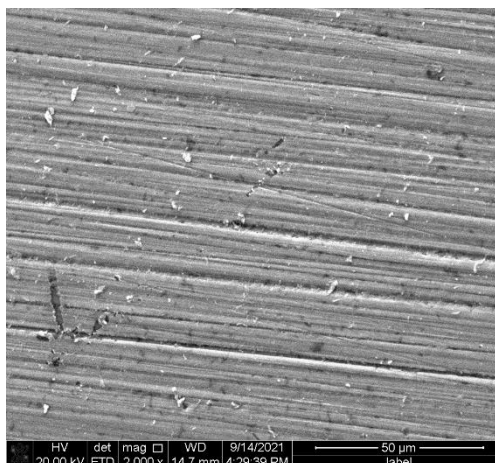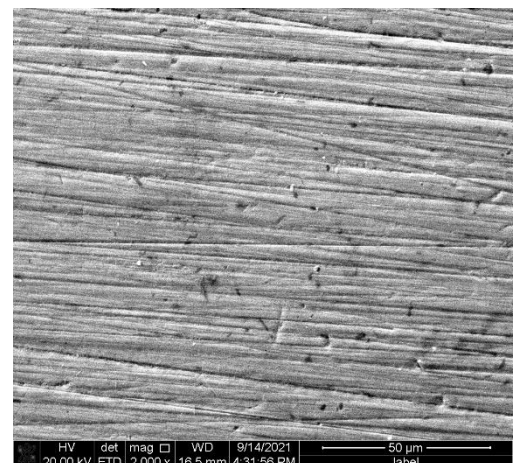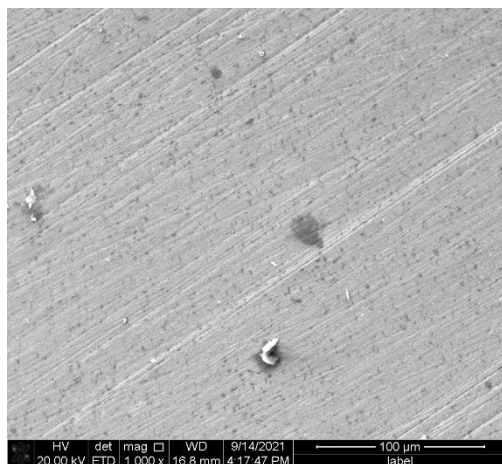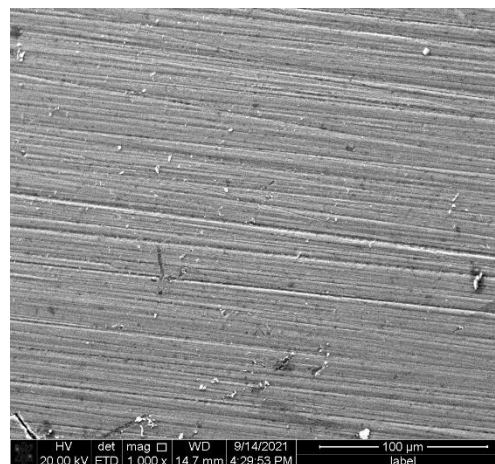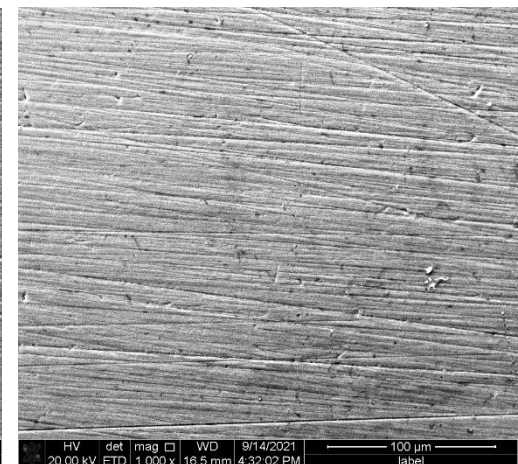

Gas Flow

1

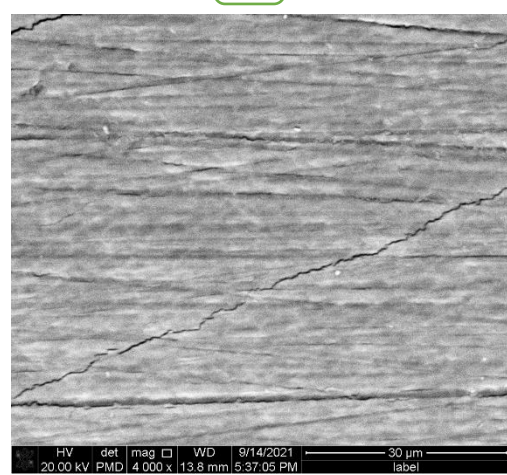

2

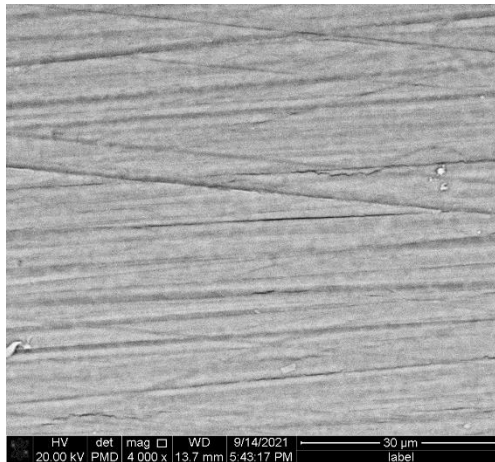

3

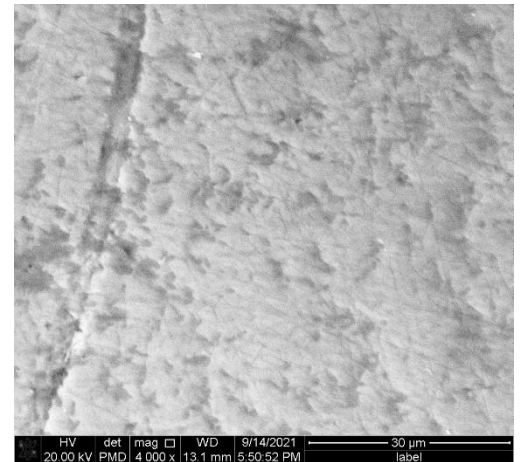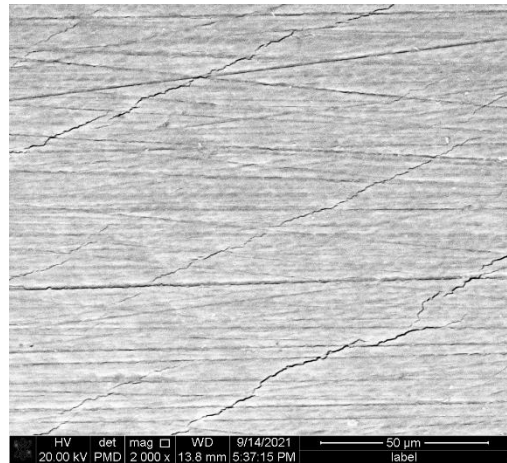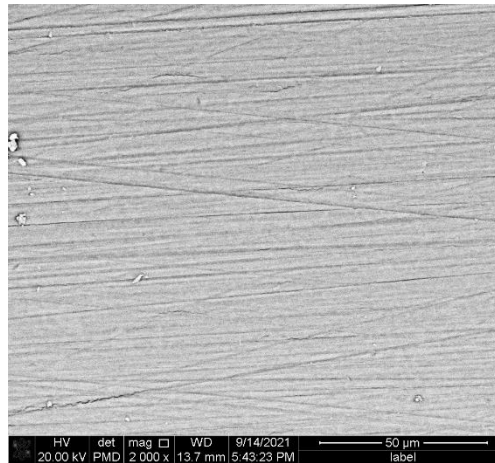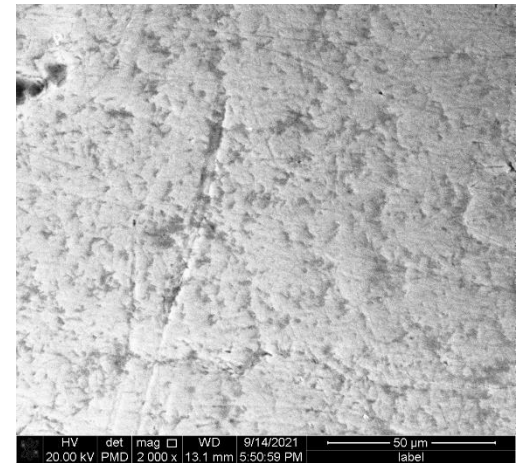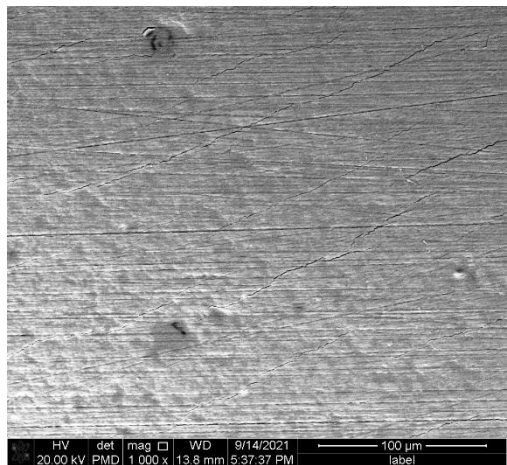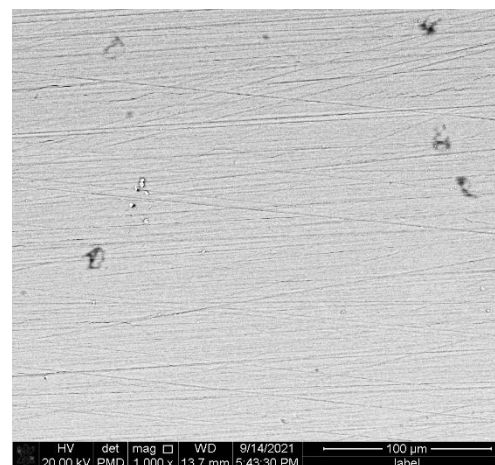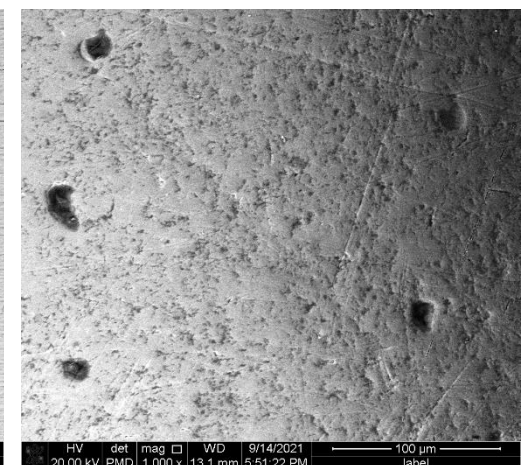

Control

Supplement: Supplementary file 1 [file materials-18-04466-s001.zip › Raw Result/SEM/SEM Pic.pdf]
